# miRNA-ome plasma analysis unveils changes in blood–brain barrier integrity associated with acute liver failure in rats

**DOI:** 10.1186/s12987-023-00484-7

**Published:** 2023-12-08

**Authors:** Karolina Orzeł-Gajowik, Krzysztof Milewski, Magdalena Zielińska

**Affiliations:** 1 Department of Neurotoxicology, Mossakowski Medical Research Institute, Polish Academy of Sciences, Pawińskiego St. 5, 02-106 Warsaw, Poland; 2 Laboratory of Cellular Metabolism, Nencki Institute of Experimental Biology, Polish Academy of Sciences, Pasteura St. 3, 02-093 Warsaw, Poland

**Keywords:** Acute liver failure, Hyperammonemia, Blood–brain barrier, Micro RNA

## Abstract

**Background:**

Hepatic encephalopathy (HE) symptoms associated with liver insufficiency are linked to the neurotoxic effects of ammonia and other toxic metabolites reaching the brain via the blood–brain barrier (BBB), further aggravated by the inflammatory response. Cumulative evidence documents that the non-coding single-stranded RNAs, micro RNAs (miRs) control the BBB functioning. However, miRs’ involvement in BBB breakdown in HE is still underexplored. Here, we hypothesized that in rats with acute liver failure (ALF) or rats subjected to hyperammonemia, altered circulating miRs affect BBB composing proteins.

**Methods:**

Transmission electron microscopy was employed to delineate structural alterations of the BBB in rats with ALF (thioacetamide (TAA) intraperitoneal (ip.) administration) or hyperammonemia (ammonium acetate (OA) ip. administration). The BBB permeability was determined with Evans blue dye and sodium fluorescein assay. Plasma MiRs were profiled by Next Generation Sequencing (NGS), followed by in silico analysis. Selected miRs, verified by qRT-PCR, were examined in cultured rat brain endothelial cells. Targeted protein alterations were elucidated with immunofluorescence, western blotting, and, after selected miR mimics transfection, through an in vitro resistance measurement.

**Results:**

Changes in BBB structure and increased permeability were observed in the prefrontal cortex of TAA rats but not in the brains of OA rats. The NGS results revealed divergently changed miRNA-ome in the plasma of both rat models. The in silico analysis led to the selection of miR-122-5p and miR-183-5p with their target genes occludin and integrin β1, respectively, as potential contributors to BBB alterations. Both proteins were reduced in isolated brain vessels and cortical homogenates in TAA rats. We documented in cultured primary brain endothelial cells that ammonia alone and, in combination with TNFα increases the relative expression of NGS-selected miRs with a less pronounced effect of TNFα when added alone. The in vitro study also confirmed miR-122-5p-dependent decrease in occludin and miR-183-5p-related reduction in integrin β1 expression.

**Conclusion:**

This work identified, to our knowledge for the first time, potential functional links between alterations in miRs residing in brain endothelium and BBB dysfunction in ALF.

**Supplementary Information:**

The online version contains supplementary material available at 10.1186/s12987-023-00484-7.

## Introduction

Hepatic encephalopathy (HE) is a debilitating neuropsychiatric condition resulting from impaired liver function. Clinical manifestation of HE ranges from mild psychiatric and neurological disturbances in chronic HE, and, brain edema often leading to coma at the acute HE stage, making the disease a significant threat to the healthcare system [1, 2]. The pathophysiology of HE encompasses brain intoxication as a result of inefficient blood clearance of a plethora of toxic when are in excess, metabolites (i.e., ammonia, aromatic amino acids, mercaptans, manganese, benzodiazepines, or xenobiotics) [3]. Accumulated data also strengthens a synergy of inflammation in HE pathology, leading to disease progression [4] beyond the sole and dominant role of harmful ammonia.

The blood–brain barrier (BBB) controls the exchange of diverse biological substances from the blood to the brain. The BBB disruption, particularly changes in junctional proteins’ structures composed of tight junction (TJ) proteins, integrins, annexins, etc., is a directly documented pathological event promoting cerebral edema, a leading cause of a fatal outcome in acute HE [5–7]. Structural BBB abnormalities were reported in the cerebral cortex of patients who died due to ALF [8], but also in patients suffering from chronic liver insufficiency, namely liver cirrhosis [9]. While morphological changes of BBB reported in ALF animal models are significant but similar to what was observed in *post-mortem* human tissues, available results differ regarding the BBB failure characteristics, referring to animal species, implemented HE model (toxins/surgical procedure), and severity related to the HE duration, as critical factors of reported diversity of BBB changes, the molecular mechanisms contributing to BBB impairment, are still lacking many details. The BBB disturbances encompassing decomposition of BBB structural elements, uncontrolled influx of different substances thru transporters/channels leading to increased BBB permeability were reported in most of reproducible ALF animal models. Inconsistencies were noticed in a grade of BBB disturbances and BBB structural proteins (e.g., zonula occludens 2, occludin, claudin 5) changes which implicate that alterations may even precedes permeability changes, suggesting that TJ disruption might occur before BBB permeability changes. Additionally, studies published till now mostly focused on astrocytes and astrocytic swelling while other cell types such as the endothelial cells and pericytes, composing BBB in the brain are involved in the pathogenesis of HE. Additionally, at the molecular level, mechanistic details of TJ elements disruption involving microRNAs (miRs) action is yet to be determined.

MiRs, small non-coding RNA sequences, can negatively regulate protein levels by suppressing gene expression. Emerging evidence has suggested circulating non-coding RNAs’ diagnostic and therapeutic values in pathologies linked with liver diseases, including ALF [10, 11]. Various miRs were recently proposed as possible biomarkers and regulatory factors in numerous CNS disorders expanding miRs’ contributing role in the control of the functioning of BBB-composing endothelial cells [12–14].

In this study, we hypothesize that BBB dysfunction in rat models of ALF results at least in part from an altered pattern of circulating miRs and that alterations affecting specific miRs impact proteins engaged in BBB integrity. To verify this, we performed Next Generation Sequencing (NGS) of plasma in two rat models: (i) ALF induced by the ip. administration of hepatotoxin thioacetamide (TAA) and (ii) primary hyperammonemia, induced by ammonium acetate (OA) ip. injection. Notably, both models present elevated plasma ammonia concentrations, as a common denominator. While the OA model is beneficial for investigating hyperammonemia, it may not completely imitate the acute nature of the illness found in patients. In turn, TAA-induced liver injury frequently causes a twofold increase in blood ammonia levels [15, 16] and generates a variety of systemic and neuroinflammatory alterations.

We assessed our hypothesis using two approaches. The first, descriptive, comprises the examination of the BBB ultrastructure by transmission electron microscopy (TEM) and evaluation of the BBB permeability to low, and high-weight markers. Further, in silico identification and selection of specific genes regulated by established and validated miRs, was followed by the analysis of identified targets’ biological function. The second approach was mechanistic and aimed at analyzing the phenotype of cultured endothelial cells (rat brain endothelial cell line and isolated brain microvascular endothelial cells) and their functioning upon treatment with ammonia and/or TNFα, the key factors involved in the pathomechanism of HE.

## Methods

### Animal models and biochemical analysis

**Scheme 1:**
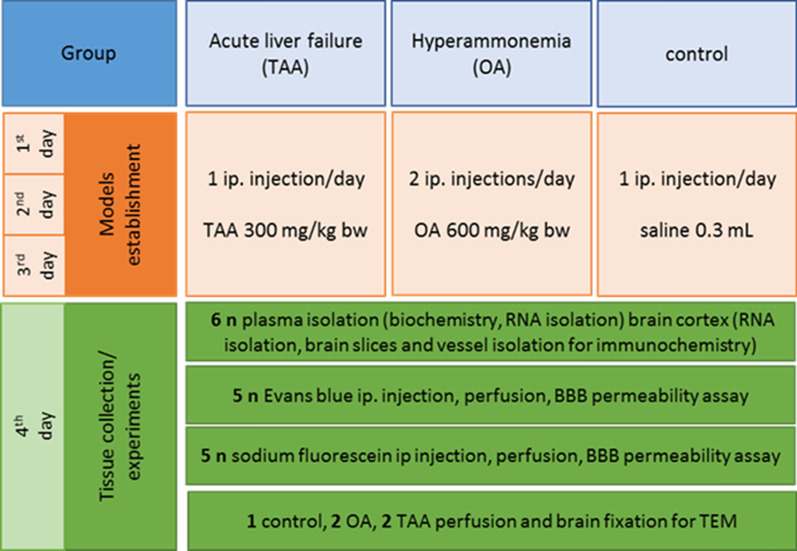
Graphical representation of the research design

Fifty-three 8–10-week-old male Sprague Dawley rats with an initial body weight of 250–300 g, from the outbred animal colony re-supplied by the Animal House of Mossakowski Medical Research Institute, Warsaw, Poland (Approval No. 57/2015 (14 May 2015 from the 4th Local Ethics Committee for Animal Experimentation, Warsaw, Poland, as compliant with Polish Law) were randomized to the experimental groups (Scheme 1). All efforts have been made to reduce the number of animals and minimize their suffering. The study complies with the ARRIVE (Animal Research: Reporting In Vivo Experiments) guidelines (study design, experimental procedures, housing and husbandry, statistical methods) guidelines for reporting animal research. Animals were given free access to water and standard rodent chow and housed in constant temperature, humidity, and 12 h light-dark cycling.

Acute liver failure (ALF) was induced by thioacetamide (TAA) intraperitoneal (ip.) injections in doses of 300 mg per kg at 24-h intervals for 3 days. The control (sham) group received 0.3 mL of 0.9% NaCl. Hyperammonemia was induced by ip. administration of ammonium acetate (OA) in a dose of 600 mg per kg at 12 h intervals for 3 days. Rats were sacrificed by decapitation and the brains were quickly removed, and the cerebral cortex was isolated. The blood was collected in EDTA tubes, incubated at room temperature for 20 min, and centrifuged at 1500×*g* for 10 min at room temperature for plasma separation.

The blood plasma ammonia level and liver enzymes (aspartate aminotransferase; AST, alanine aminotransferase; ALT, and γ-glutamyl transpeptidase; GGTP) activities were measured by a commercial analyzer (Scil Vet, Germany). Levels of proinflammatory cytokines were evaluated using LEGENDplex™ Rat Inflammation Panel (BioLegend, San Diego, USA) directly according to manufacturer instructions. Data acquisition (300 events for each cytokine) was performed using a BD FACSCanto II flow cytometer with BD FACSDiva Software and FCAP Array software, version 3.0 (BD Biosciences, San Jose, USA).

### Rat brain capillaries isolation

After decapitation, brains were extracted, cerebral cortices isolated, and homogenized in Ringer’s solution then centrifuged at 1500×*g* for 10 min at 4 °C. The pellet was re-suspended in fresh buffer and centrifuged; the washing step was repeated three times under the same conditions. The final pellet was homogenized in 10 mL of 0.25 M sucrose and centrifuged in a discontinuous sucrose gradient (0.25:1:1.5 M sucrose) (30,000×*g*, 30 min, and 4 °C). The fraction with microvessels was collected and transferred to the Eppendorf tube.

### Cell cultures and miRs’ transfection

Primary brain microvascular endothelial cells (PBMEC) were isolated from the vesicles of the brain cortex of 14-day-old Sprague-Dawley male rats from the animal colony of Mossakowski Medical Research Institute, Polish Academy of Sciences, directly according to the protocol [17]. Briefly, after decapitation, brains were isolated, and cortices tissue was dissected under sterile conditions. The homogenized tissue was enzymatically and mechanically dissociated with 1 mL of 10 × Trypsin and Type IV Collagenase solution at 37 °C for ~ 30 min. Obtained pellets after following centrifugation and cleaning steps (800×*g* for 5 min, ~ 20 °C; 1500×*g* for 15 min ~ 20 °C; 800×*g* for 15 min), and the supernatant was discarded. Cells were seeded on a 6-well plate coated with collagen I (Corning, NY, USA) and cultured in Dulbecco’s Modified Eagle’s Medium (DMEM) containing 20% FBS and supplemented with 1.0 ng/mL basic fibroblast growth factor (bFGF) and 20.0 µg/mL bovine sodium heparin. In the first 2 days, puromycin was added to the culture medium to inhibit neuron and astrocyte growth. The medium was changed the next day, and then every 2–3 days.

Rat brain endothelial cells (RBE4) were cultured in 24-transwell plates, 60 mm, or 100 mm dishes (Corning, NY, USA) coated with collagen type I. Cells were cultured on the MEM/Ham’s F10 medium with Glutamax (Gibco, Thermo Fisher Scientific, Waltham, USA) with the addition of 10% of heat-inactivated FBS, 1.0 ng/mL bFGF, and gentamicin (Gibco, Thermo Fisher Scientific, Waltham, USA) at 37 °C in a humidified atmosphere of 95% air and 5% CO_2_ and used after 14–21 days when cells displayed endothelial phenotype and minimum 90% of the surface confluence.

The PBMEC and RBE4 cells were treated with 5 mM ammonium chloride (“ammonia”) or 10 ng/mL TNFα (rat recombinant, Sigma-Aldrich, Saint Louis, USA) which were added into the cell culture medium for 24 h.

For transfection experiments, RBE4 cells were seeded at density 3,3 × 10^5^ and were transfected with the following sequences: 5′-UGGAGUGUGACAAUGGUGUUUG-3′ for mimic miR-122-5p (Qiagen; Cat. No. 33173YM00470430-ADA) and 5′-UAUGGCACUGGUAGAAUUCACU-3′ for mimic miR-183-5p (Qiagen; Cat. No. 339173YM00471390-ADA) or negative control 5′-UCACCGGGUGUAAAUCAGCUUG-3′ (Qiagen; Cat. No. 339173YM00479902-ADA). Transfection was performed using the HiPerFect transfection reagent (Qiagen, Hilden, Germany) according to the manufacturer’s instructions, using 5 µM or 20 µM mimic concentration for 24 h. For cell adhesion experiments RBE4 cells were seeded at density 1 × 10^5^ on a 6-well plate coated with collagen I.

### RNA extraction and next generation sequencing

Total RNA was extracted from 200 µL rat plasma using the miRNeasy Serum/Plasma Advanced Kit (Qiagen, Hilden, Germany). Samples were thawed on ice and centrifuged at 12,000×*g* for 5 min to remove any cellular debris. For each sample, 200 µL of plasma was mixed with 1 mL of Qiazol followed by extraction steps directly according to the manufacturer’s instructions. Finally, RNA was eluted in 10 µL of nuclease-free water and stored at − 80 °C to await further analysis. Quantification of total RNA was made spectrophotometrically with a NanoDrop 2000 (Thermo Fisher Scientific, Waltham, USA) and 1 µg of total RNA was used for library preparation. NGS procedure was carried out by the BGI company (Shenzhen, China) using DNBSEQ G400 (MGI2000) system in SE50 mode. A minimum of 20 million paired-end reads were generated per sample. Quantification includes the raw read count, as well as normalized expression level as CPM values (counts per million reads mapped) to account for the variability in the library size. Next, miRDeep2 Quantifier analysis based miRbase 2.0 library was used to identify miR sequences. The quantification of the miR expression level was carried out using the Quantifier script of the miRDeep2 tool applying the default parameters. MiRs with a false discovery rate (FDR) p-value determined by DESeq2 below 0.05 were considered as differentially expressed (up/downregulated).

### Quantitative RT-PCR

Reverse transcription and cDNA synthesis were performed using TaqMan Advanced miRNA cDNA Synthesis Kit (Invitrogen, ThermoFisher Applied Biosystems™, A28007) according to the manufacturer’s protocol. The levels of miR expression were measured by quantitative RT-PCR with a miRCURY LNA SYBR Green PCR Kit (Qiagen, Hilden, Germany, Cat No. 339345) with appropriate miRCURY LNA miRNA PCR Assay primers: rno-miR-183-5p (Qiagen, Hilden, Germany, Cat No. QG-339306_YP00206030) and rno-miR-122-5p (Qiagen, Hilden, Germany Cat No. QG-339306_YP00205664). The snRNA U6 was used as a normalization control (Qiagen, Hilden, Germany Cat No. QG-339306_YP02119464). The assay was conducted using the Applied Biosystems 7500 Sequence Detection System (Applied Biosystems, Foster City, CA, USA) according to the manufacturer’s instructions. The relative expression levels of miRNAs were calculated using the 2^−ΔΔCt^.

### Western blot analysis

To evaluate the occludin and integrin β1 protein content in rat cerebral cortex Western blot analysis was used. Triton Lysis Buffer (20 mM Tris pH 6.8, 137 mM NaCl, 2 mM EDTA, 1% Triton X-100, 0.5 mM DTT, 1 mM PMSF) containing Protease Inhibitor Cocktail (Sigma-Aldrich, Germany) and Phosphatase Inhibitor Cocktail (Sigma-Aldrich, Germany) was used to homogenize brain tissue. The homogenate was centrifuged at 12,000×*g* for 20 min at 4 °C. On a 10% SDS-polyacrylamide gel, equal amounts of protein (30 µg) were separated and deposited onto a nitrocellulose membrane. After overnight incubation with an anti-occludin antibody (1:200, Cell Signalling, Inc., Cat. No. 91131) or integrin β1 (1:200, Cell Signalling, Inc., Cat. No. 4706) the membranes were washed, treated with HRP-conjugated anti-rabbit IgG (1:2000; Sigma-Aldrich, Germany), and developed with West-Pico Chemiluminescence Substrate (Pierce, Rockford, USA). After stripping, the blots were treated for 1 h with an anti-GAPDH antibody.

### Bioinformatics analysis

To evaluate the biological impact of the differentially expressed miRs on target genes, we used three different databases: miRDB v5 (http://mirdb.org/miRDB, TargetScan7.1 (https://www.targetscan.org/vert_80/), and TarBase v.8 (https://dianalab.e-ce.uth.gr/html/diana/web/index.php?r=tarbasev8%2Findex). We selected a set of targets linked to BBB structure or function, repetitively reported in all three databases. Next, based on the miRDB tool MirTarget, we predict the most probable miR target genes (miRs with targets with a score < 60 were not taken into further analysis according to miRDB authors’ recommendations). Next, we associated data with NGS results considering differently expressed miRNAs but with the lowest p-value, resulting in the most altered miRs with target genes of the highest probability.

After establishing specific genes regulated by selected miRs, we identified the potential of selected genes on biological functions. The String Tool pathways analysis was performed to verify the probable contribution of selected genes to a broader spectrum of processes. We examined the involvement of selected genes in the biological processes as well as KEGG Pathways in combination with gene clustering. The String Tool pathways analysis showed interactions with a confidence score greater than the minimum needed (0.15) as a result only those networks are included in the prediction. The probability that a predicted link between proteins in the same metabolic map in the KEGG database is represented by the confidence score. Following this, we set the required interaction score of 0.9 the highest confidence interaction.

### Immunohistochemical and immunofluorescent staining

RBE4 and PBMEC cells were seeded onto collagen I-coated coverslips at a density of 3 × 10^2^ cells/well and cultured for 5–7 days. Cells were fixed with 4% paraformaldehyde/PBS and permeabilized with 0.25% Triton X-100/PBS. Secondly, were incubated with antibodies against CD-31 (1:400, ab28364 Abcam, Cambridge, UK), vWF (1:400, ab6994, Abcam, Cambridge, UK) overnight at 4 °C and then exposed to secondary antibody Alexa Fluor 488 goat anti-rabbit IgG (H+L) (1:1000, Thermo Fisher Scientific, Waltham, MA, USA, Cat. No. A11008).

After 24 h incubation with ammonia and/or TNFα, cells were rinsed with PBS. Cultured cells, brain cortex slices, and isolated brain microvessels were fixed with 4% paraformaldehyde/PBS and permeabilized with 0.25% Triton X-100/PBS. Secondly, were incubated with antibodies against occludin (1:200, Cell Signalling, Inc., Cat. No. 91131) or integrin β1 (1:200, Cell Signalling, Inc., Cat. No. 4706) and CD-31 (1:400, ab28364 Abcam, Cambridge, UK) overnight at 4 °C, and then exposed to secondary antibody Alexa Fluor 488 goat anti-rabbit IgG (H+L) (1:1000, Thermo Fisher Scientific, Waltham, MA, USA, Cat. No. A11008) and Alexa Fluor 546 goat anti-rabbit IgG (H+L) (1:1000, Thermo Fisher Scientific, Waltham, MA, USA, Cat. No. A11008). The nuclei were labeled with Hoechst 33258 (Thermo Fisher Scientific, Waltham, MA, USA, Cat. No. H1398). Images were acquired in a confocal laser scanning microscope LSM780 (Zeiss, Jena, Germany) and processed using the ZEN 2012 (Zeiss, Jena, Germany). To quantify fluorescence intensity, the pictures were subjected to a scaling process to achieve a magnification factor of 20. Additionally, the images were normalized to ensure consistent exposure duration across all samples. A total of eight to ten regions of interest were quantified using ImageJ (Fiji) software, with background fluorescence subtracted from both control and examined sections.

### Transendothelial electrical resistance and cells adhesion

The transendothelial electrical resistance (TEER) was measured by a custom-made device, developed for in vitro experiments. RBE4 cells were seeded at a density of 6 × 10^5^, cultured in transwell chambers with 0.4 mm polycarbonate filters (Corning Costar, Corning, NY, United States) at 37 °C in a humidified atmosphere of 95% air and 5% CO_2_. Before treatment with the ammonia and TNFα, TEER was measured every 5 min. for 1 h in all inserts. After the treatment, the TEER in 24-well was measured every 5 min. for 24 h. Each day of the experiment was followed by the medium exchange. TEER value was expressed in Ω, and calculated by subtracting the resistance of a transwell without cells from a well with cells.

Using a custom-made device cell adhesion to collagen I was evaluated using the adapted methodology described by Lajkó et al. [18]. The adhesion was measured in 4-h time intervals. The Ω values were obtained as a variation of the initial values of seeded cells to values within 24 h of cell attachment. Results were normalized to uncoated transwell.

### Evans blue and sodium fluorescein permeability assay

2% Evans blue (EB; Sigma-Aldrich, Saint Louis, USA) in sterile saline was injected into the tail vein in rats (4 mL/kg. After 2 h circulation, rats were transcardially perfused with 200 mL of ice-cold saline, and the brains were quickly removed. The isolated brain cerebral cortices were weighed, homogenized in 30% trichloro acid (TCA), and centrifuged at 10,000×*g* for 20 min. The absorbance of the supernatants was measured at 620 nm, using a Pharmacia LKB Ultrospec III spectrophotometer (Uppsala, Finland), and BBB permeability was calculated as a fluorescent intensity. Results were calculated using EB as a standard and expressed as nanograms per milligram of tissue brain tissue. Sodium fluorescein (SF; 100 mg in 1 ml saline; Sigma-Aldrich, Saint Louis, USA) was injected ip., and after 45 min rats were perfused with saline. After decapitation, cerebral cortices were removed, weighed, and homogenized 1:10 (w/v) in sterile PBS. Samples were precipitated with ethanol (1:3 v/v) followed by centrifugation at 3000×*g* for 10 min. The supernatants were diluted and analysed in a spectrofluorometer FLUOstar Omega (BMG Labtech, Ortenberg, Germany) using an excitation wavelength of 480 nm and an emission wavelength of 538 nm. The BBB permeability was measured as the ratio of SF in a gram of brain tissue to the amount of SF in 1 ml of plasma and present in a percentage.

### Transmission electron microscopy

One control, two OA, and two TAA rats were anesthetized and perfused through the ascending aorta with 2% paraformaldehyde and 2.5% glutaraldehyde in 0.1 M cacodylate buffer, pH 7.4. The cerebral cortex was fixed in the same solution for 20 h (at 4 °C) and placed in 1% OsO_4_ for 6 h. Afterward, the samples were dehydrated by ethanol solutions of increasing concentration (30%–10 min, 50%–10 min, 70%–24 h, 90%–10 min, 96%–10 min, anhydrous EtOH–10 min, finally acetone–10 min) and saturated with epone. Epone was polymerized in blocks at 60 °C for 24 h in an incubator (Agar Scientific, Stansted, England). The polymerized samples were cut into ultrathin sections (70 nm thick) with an RMC MTX ultramicrotome (Boeckeler Instruments, Tucson, Arizona, USA), placed onto copper nets, and analyzed in a LIBRA 120 transmission electron microscope (Zeiss, Oberkochen, Germany). Photographs were taken with a Slow-Scan CCD camera (ProScan, Germany), using the EsiVision Pro 3.2 software. We evaluated 12–24 fields of view from each sample.

### Statistical analysis

Data were analyzed and visualized using Prism 7 (GraphPad Software Inc., La Jolla, USA). Results were presented as mean ± SD. The statistical significance between various groups or treatments was measured by unpaired t-test or one-way ANOVA with Dunnett’s post hoc test. In all experiments, p-value < 0.05 was considered to be statistically significant, ***p < 0.001, **p < 0.01, *p < 0.05, without asterisks means no significance.

## Results

### TAA treatment generates severe hepatic injury

Both animal models were previously described and are routinely used in our laboratory and by other groups to study different aspects of HE [19–21]. The levels of ALT and AST were significantly increased in the TAA group acknowledging liver injury. Ammonia levels were markedly increased in both OA and TAA rats (Table 1). Determination of systemic inflammation revealed notable inter-individual variation in the plasma cytokines level. Significantly increased plasma levels of Il-6, IL-10, and Il-1β were measured in TAA rats. Enhanced TNFα levels, noticed in 50% of the TAA group of animals, presented a tendency toward an increase. In turn, the OA group did not present marks of inflammation except for the elevation of Il-1β (Table 1).

**Table 1:** Biochemistry and cytokines concentration in rat plasma

	Control	OA	TAA
Ammonia (µmol/L)	62 ± 7	93 ± 11**	139 ± 11***
AST (units/L)	163 ± 17	175 ± 47	1958 ± 851***
ALT (units/L)	64 ± 6	70 ± 10	442 ± 154***
GGTP (units/L)	1.7 ± 0.3	1.9 ± 0.7	2.1 ± 0.4
TNFα (pg/mL)	0.039 ± 0.103	0.009 ± 0.024	5.435 ± 10.150
IL-1α (pg/mL)	4.970 ± 7.678	6.034 ± 4.177	59.648 ± 42.246
IL-1β (pg/mL)	n.d.	0.427 ± 1.131***	1.874 ± 6.215***
IL-6 (pg/mL)	95 ± 106	n.d.	10,022 ± 1355***
IL-10 (pg/mL)	n.d.	n.d.	1592 ± 3313***

Results are mean ± SD. n = 6; **p *<* 0.01, ***p *<* 0.001 vs. control

### Differences and similarities of structural and functional changes of the BBB induced by TAA and OA

**Fig. 1:**
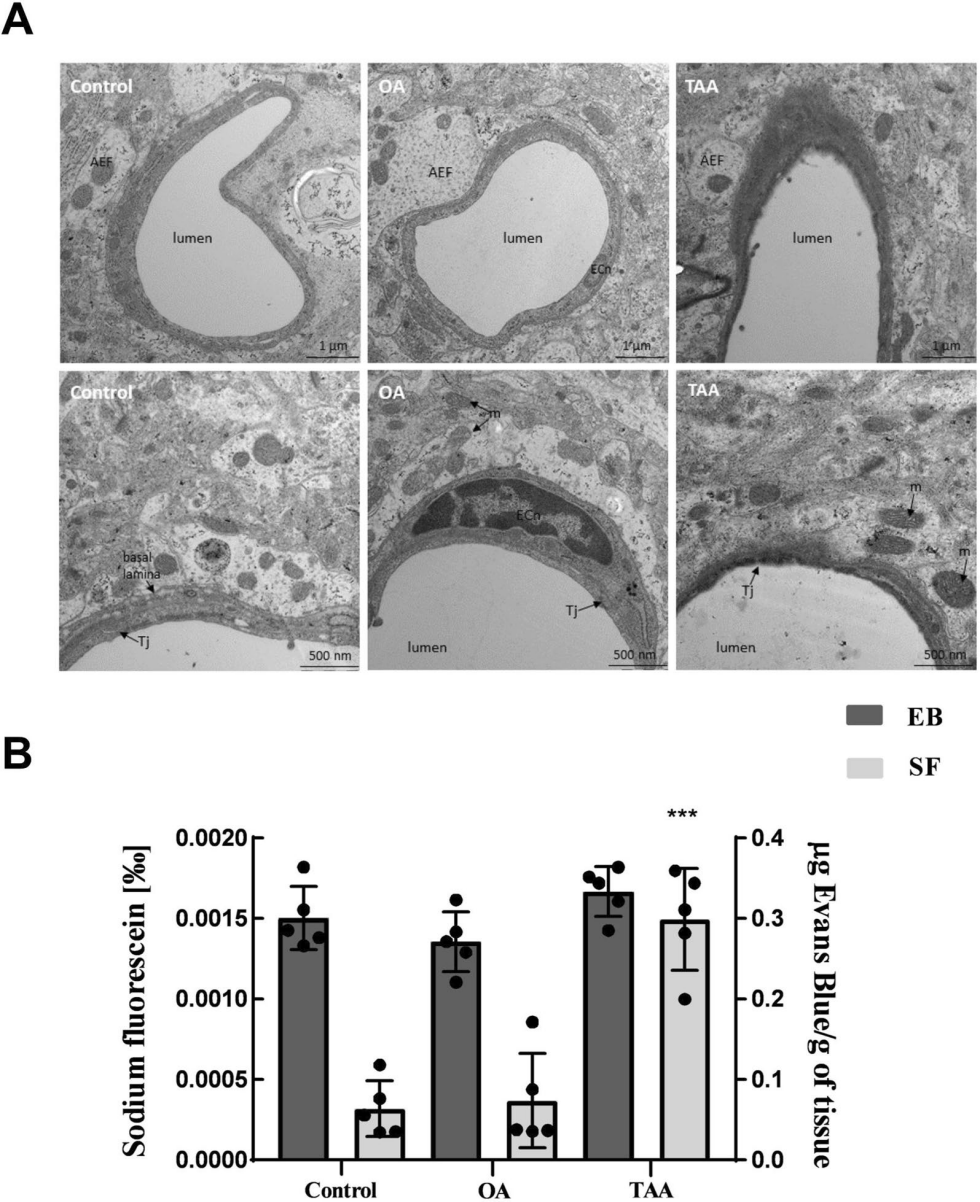
The effect of OA and TAA administration on the rat BBB structure and permeability. **A** TEM images of the microvessels in rat brain prefrontal cortex of the control, OA, and TAA rat. *j* tight junctions, *m* mitochondria, *ECn* endothelial cell nuclei, *AEF* astrocyte endfeet. Scale bars are posted under individual images. **B** BBB permeability for Evans blue (EB; black bars) and sodium fluorescein (SF; grey bar). The results are the mean ± SD. n = 5; ***p < 0.0001 vs. control, one-way ANOVA, Dunnett’s post hoc test

Ultrastructural changes of the BBB were verified by TEM imaging. Unchanged microvascular endothelial cells and astrocytes with unaltered structures were observed in the control and OA rat brain prefrontal cortex (Fig. 1A; left and middle panels). In turn, endothelial cells of TAA rat brain microvessels were noticeably swollen with distinct disruption of the cytoplasmic membrane (Fig. 1A; right panel). In the peripheral zone, astrocytic endfeet with swollen mitochondria were also observed (Fig. 1A; right panel).

The BBB permeability was evaluated using a high-weight EB and a low-weight SF, dye. We did not observe any difference in the brain concentration of EB between studied experimental groups (Fig. 1B). In turn, SF concentration in the TAA brain was significantly higher, as compared to the control by ~ 500%, along with an unaltered level in the OA group (Fig. 1B). Taken together, we documented that TAA-induced ALF, unlike OA, causes structural BBB abnormalities and BBB permeability increase for low molecular weight molecules.

### Plasma miRNA expression changes differ between OA and TAA rats

We profiled miRNAs isolated from the plasma of six controls, OA, and TAA-administered rats. The NGS analyses yielded 76 × 10^6^ quality-filtered and processed reads across all samples with a mean count of 4.9 × 10^6^ per sample (Additional file 1: Tables S1, S2). The Pearson correlation analysis revealed one outlier sample in the control group which was in consequence removed from further analysis.

**Fig. 2:**
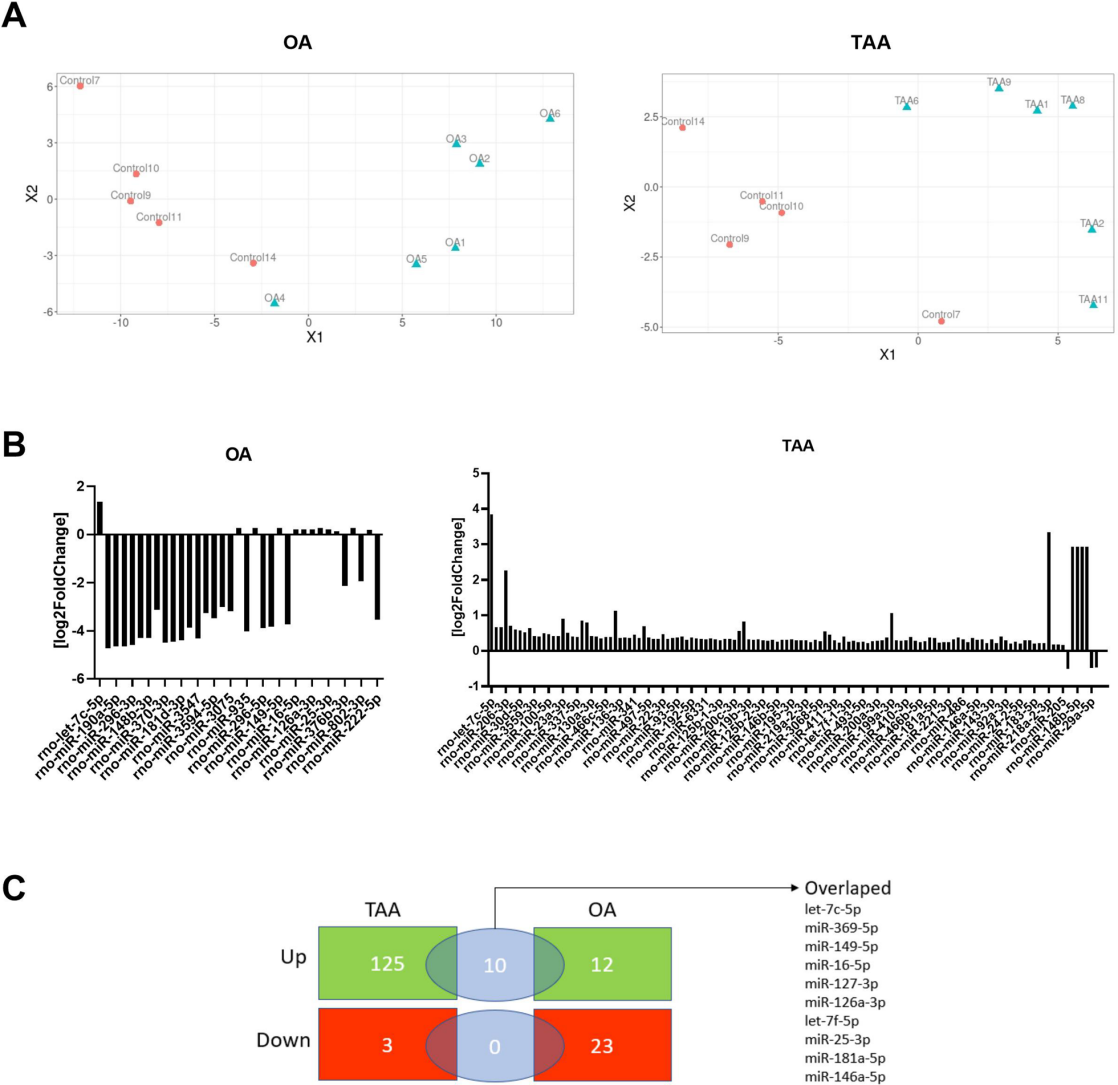
The effect of OA and TAA on miR levels in the rat plasma. **A** Multidimensional scaling plot of miR profiles of individual animals. **B** Bar plot of differentially expressed miR expressed as log2 fold changes (control vs. OA; left plot and control vs. TAA; right plot). **C** Venn diagram of differentially expressed miRNA in rat plasma showing several upregulated (green) and downregulated miRNA (red) in the studied animal models

We identified 488 circulating mature miRs across all plasma samples (Additional file 1: Tables S1, S2). Differential expression (DE) analysis revealed significant variation between experimental groups, thus in the OA group, 35 differentially expressed miR (12 upregulated, and 23 downregulated) were identified (Fig. 2C). In turn, more pronounced changes were analyzed in the TAA group: number of 128 miRs were differentially expressed, of which 125 were upregulated and 3 were downregulated (Fig. 2A). The results comparison of both models revealed high (83%) sharing of the upregulated miRNA observed in OA rats with TAA group (Fig. 2C). The results correspond well to the current knowledge that a systemic, high ammonium concentration (hyperammonemia) is a dominant factor in the course of ALF [22] reproduced well in the TAA rat model (Table 1) [20, 23]. Interestingly, in both models, the most pronounced change was miRNA let-7c-5p increase (twofold in the OA and ~ fourfold in the TAA) (Fig. 2C, Additional file 1: Tables S1, S2).

### In silico analysis of differentially expressed miRs and their target genes

**Fig. 3:**
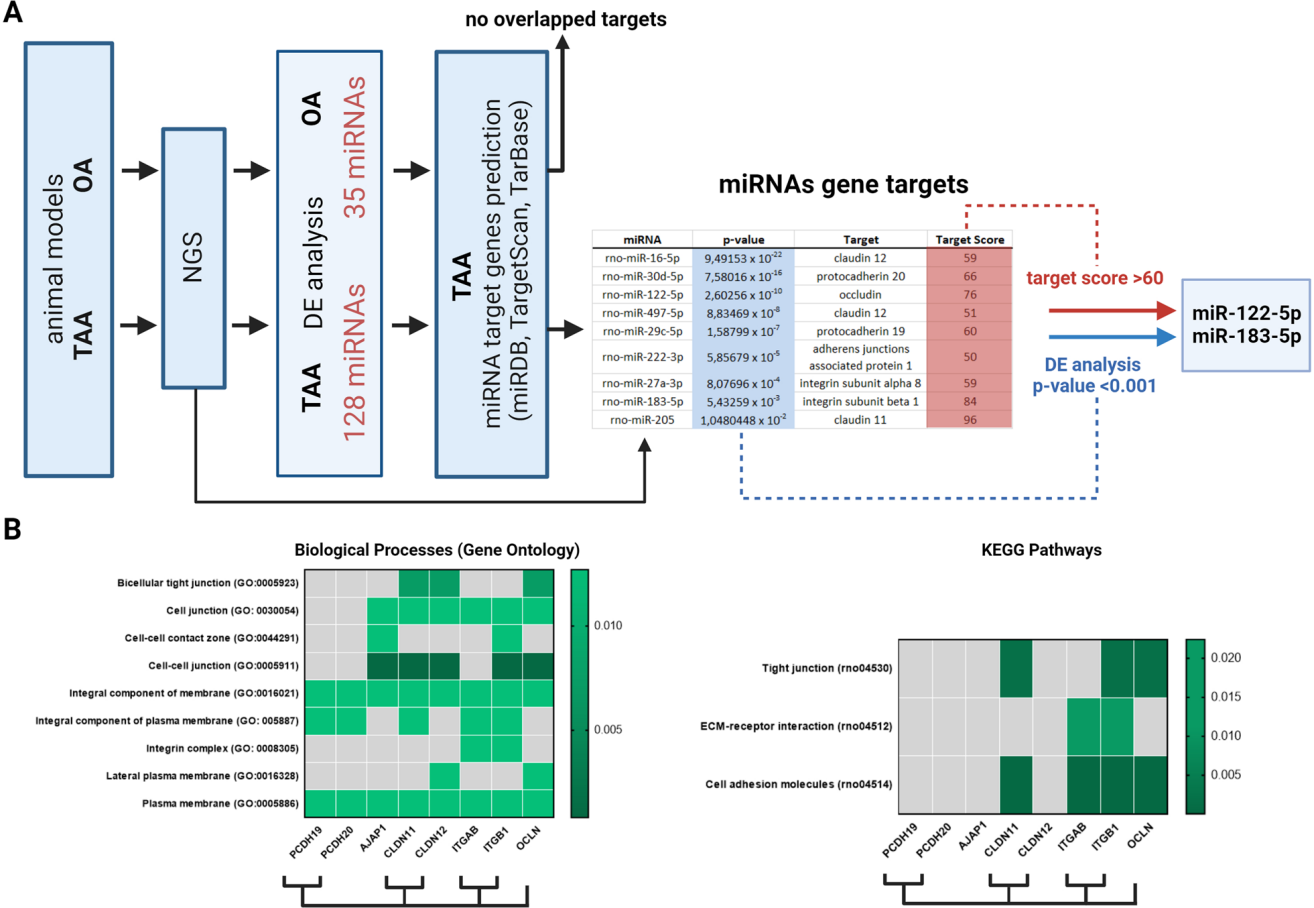
In silico analysis of differentially expressed plasma miRNA. **A** Flow chart of the miRNA target prediction analysis. **B** Heatmap presenting results of the Gene Ontology (GO) pathway analysis of nine predicted target genes (left panel). Heatmap of the KEGG Pathways analysis of nine predicted target genes (right panel)

Target genes for DE miR were predicted using three different online-available databases: miRDB, TargetScan, and TaRBase. Due to substantial differences in the obtained matches (Additional file 1: Tables S3, S4) only fully overlapped results, in all three databases, were qualified as the most relevant. As a result, nine target genes related to the BBB function were found in the TAA group (Fig. 3A, Additional file 1: Table S3), while no overlapped target was identified in the OA group (Fig. 3A, Additional file 1: Table S4). In consequence, for further analysis only TAA group was taken into consideration. Next, we evaluated the obtained data using the following criteria: low DE analysis p-value obtained in NGS (< 0.001) along with high gene target score (> 60) Displayed targets possess target prediction scores, which are rated by the new computational target prediction system. As a consequence, the search results were sorted by prediction score, additionally, rates fewer than 60 did not proceed in the study. Therefore, our analysis identified miR-122-5p and miR-183-5p as the most relevant and pointed to their potential targets: occludin and integrin β1 respectively, as the most promising for further evaluation.

We looked at the functionality of predicted gene targets by Gene Ontology (GO) analysis in terms of biological processes. Using String bioinformatics tool we identified predicted genes in nine biological processes associated with the BBB functioning: (GO:0005911 Cell-cell junction, GO:0005923 Bicellular tight junction, GO:0008305 Integrin complex, GO:0005887 Integral component of the plasma membrane, GO:0030054 Cell junction, GO:0005886 Plasma membrane GO:0016021 Integral component of membrane, GO:0016328 Lateral plasma membrane, GO:0044291 Cell-cell contact zone as well as in 3 KEGG Pathways: rno04530 Tight Junction, rno04512 ECM–receptor interaction and rno04514 Cell adhesion molecules (Fig. 3B). The occludin was associated with six GO pathways, while integrin β1with seven from nine GO pathways (Fig. 3B).

KEEG analysis selected three from nine predicted target genes (occludin, integrin β1, and claudin 11) indicating their involvement in Tight junction, ECM receptor interaction, and Cell adhesion molecules pathways (Fig. 3B).

Our results pointed out miR-122-5p and miR-183-5p with their target genes, occludin, and integrin β1, as elements potentially involved in BBB alterations observed in the TAA rat brain.

### RT-PCR analysis of miR-122-5p and miR-183-5p expression ex vivo and in vitro

**Fig. 4:**
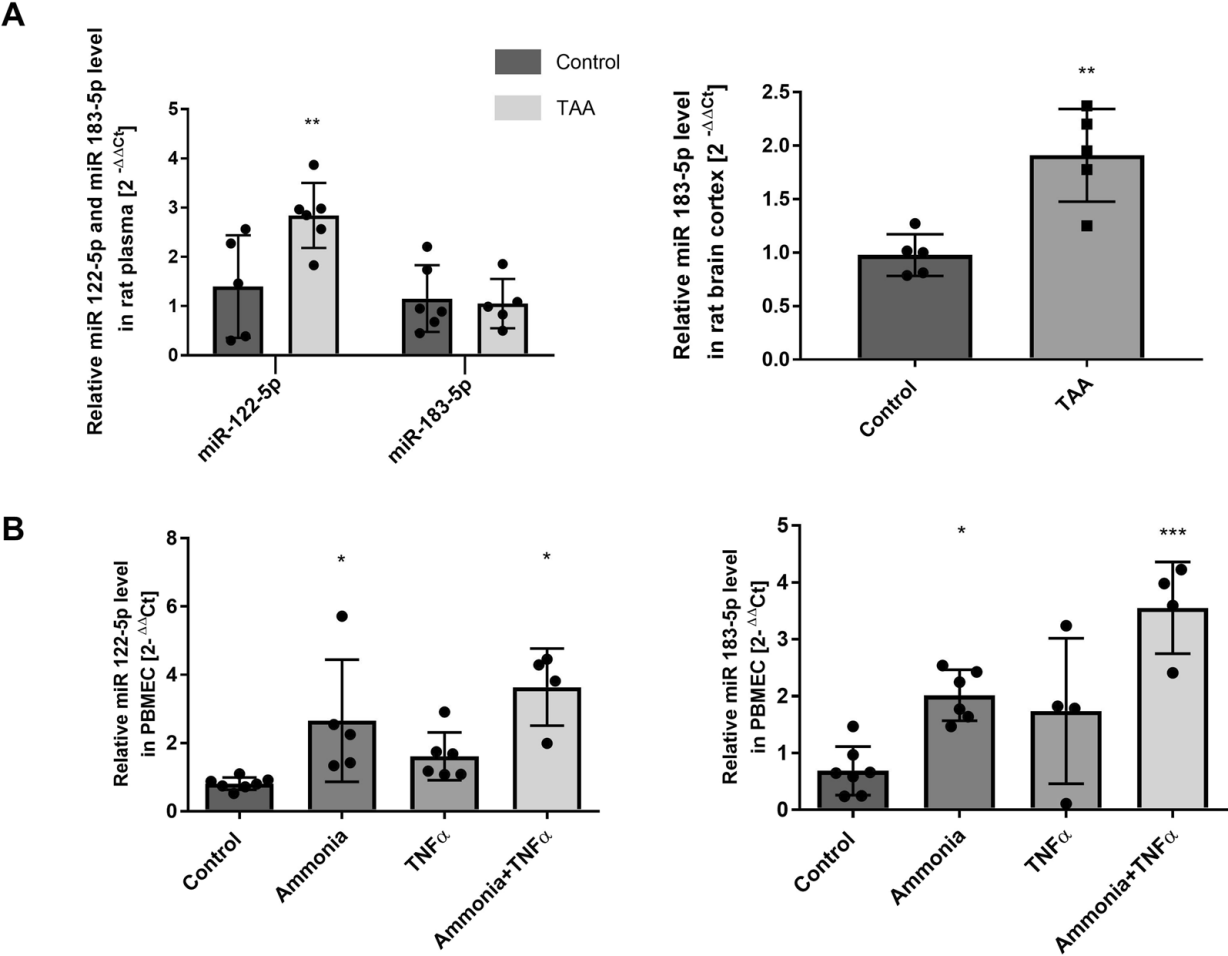
miR-183-5p and miR-122-5p expression analysis. **A** The expression of miR-122-5p and miR-183-5p in the TAA rat plasma and miR-183-5p expression in the TAA rat brain prefrontal cortex. The results are the mean ± SD. n = 5; **p < 0.001 vs. control, unpaired t-test. **B** The expression of miR-122-5p and miR-183-5p in primary rat brain microvascular endothelial cells treated with ammonia and/or TNFα. The results are the mean ± SD. n = 4; *p < 0.05 vs. control, ***p < 0.001 vs. control, one-way ANOVA, Dunnett’s post hoc test

Following the results of the NGS analysis documenting the upregulation of both miR of interest in the plasma of TAA rats, we verified their expression level using qRT-PCR analysis. A significant ~ 250% increase and unchanged expression level of the miR-122-5p and miR-183-5p, respectively, in TAA rat plasma, was observed (Fig. 4A, left panel). Since miR-183-5p is predominantly expressed in the brain (Additional file 1: Fig. S1), in parallel, we examined the miR-183-5p expression level in the prefrontal cortex of TAA rats. The revealing analysis documents the onefold increase of this miRNA in the TAA rat brain homogenate (Fig. 4A, right panel).

Next, we verified the potential of key pathogenic factors of HE (ammonia and/or TNFα) to affect miR-122-5p and miR-183-5p expression changes. Using the primary rat brain microvascular endothelial cells (PBMEC) tunnelled to mimic endothelium in vivo, first, we documented culture purity and characteristic PBMEC features that resembled fairly well native endothelium (Additional file 1: Fig. S3). We showed that ammonia, TNFα, or their combination, increase the relative expression of both miRs, with a less pronounced effect of TNFα added alone (Fig. 4B).

### The effect of TAA treatment on occludin and integrin β1 levels in the rat brain cortex homogenate and isolated brain cortex microvessels

**Fig. 5:**
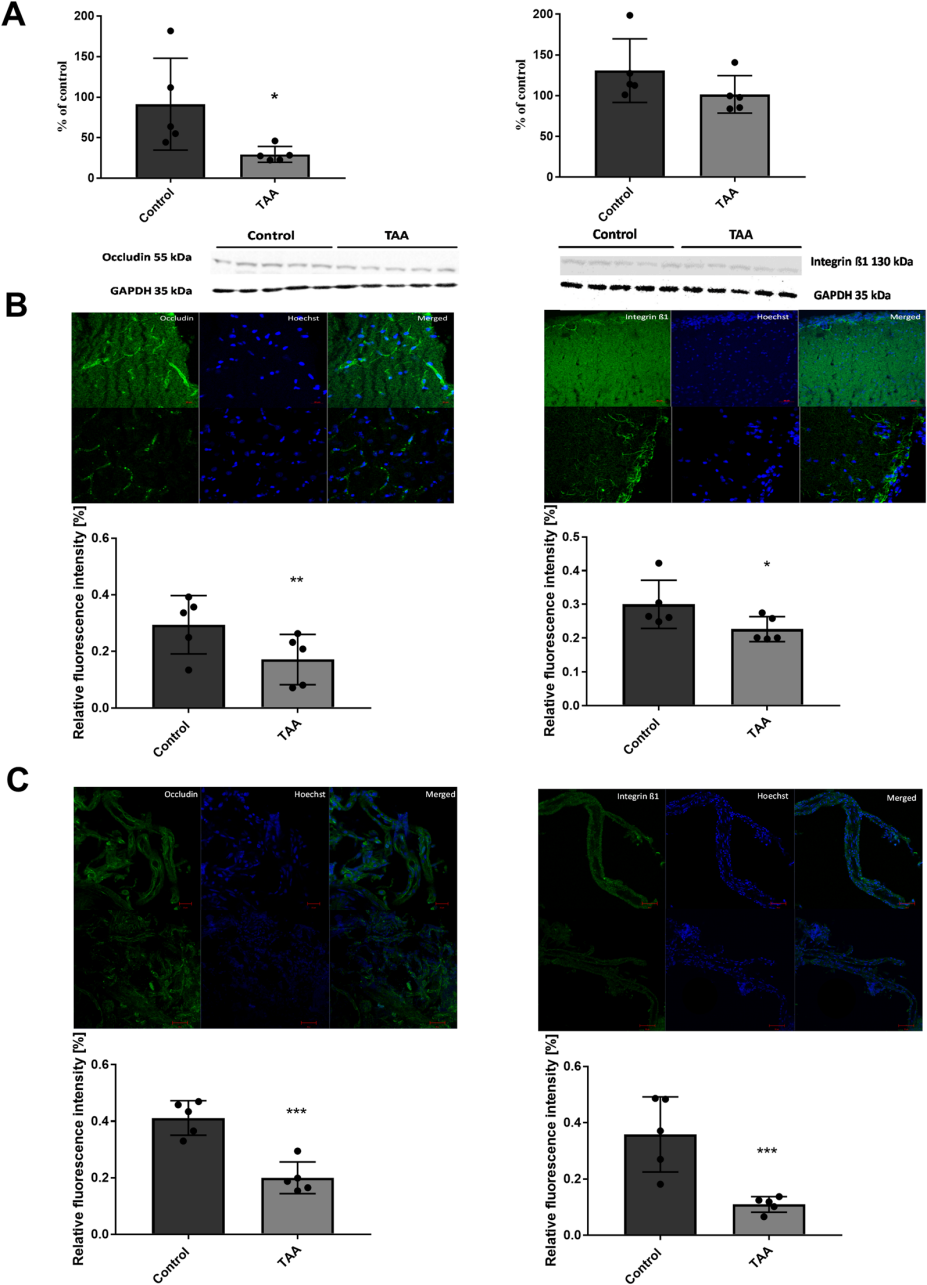
The effect of TAA treatment on occludin and integrin β1 protein level in the rat brain cortex. **A** Protein level of occludin and integrin β1 in the prefrontal cortex of TAA rat brain. The results are the mean ± SD. n = 5; *p < 0.05 vs. control, unpaired t-test. **B** The immunostaining of occludin (left panel, green) and integrin β1 (right panel, green) in isolated brain cortex, nuclei stained with Hoechst (blue). **C** The immunostaining of occludin (left panel, green) and integrin β1 (right panel, green) in brain cortex microvessels, nuclei stained with Hoechst (blue). The results are the mean ± SD. n = 5; ***p < 0.0001 vs. control, unpaired t-test

Next, we studied both proteins amount in the TAA prefrontal cortex homogenates (Fig. 5A), and immunofluorescence intensity in brain slices of the prefrontal cortex (Fig. 5B) as well as in brain microvessels (Fig. 5C) isolated from the TAA rats. A marked decrease in both occludin and integrin β1 was observed in this preparation.

### In vitro study of miR-122-5p and miR-183-5p involvement in ammonia/TNF-α-induced occludin and integrin β1 changes

**Fig. 6:**
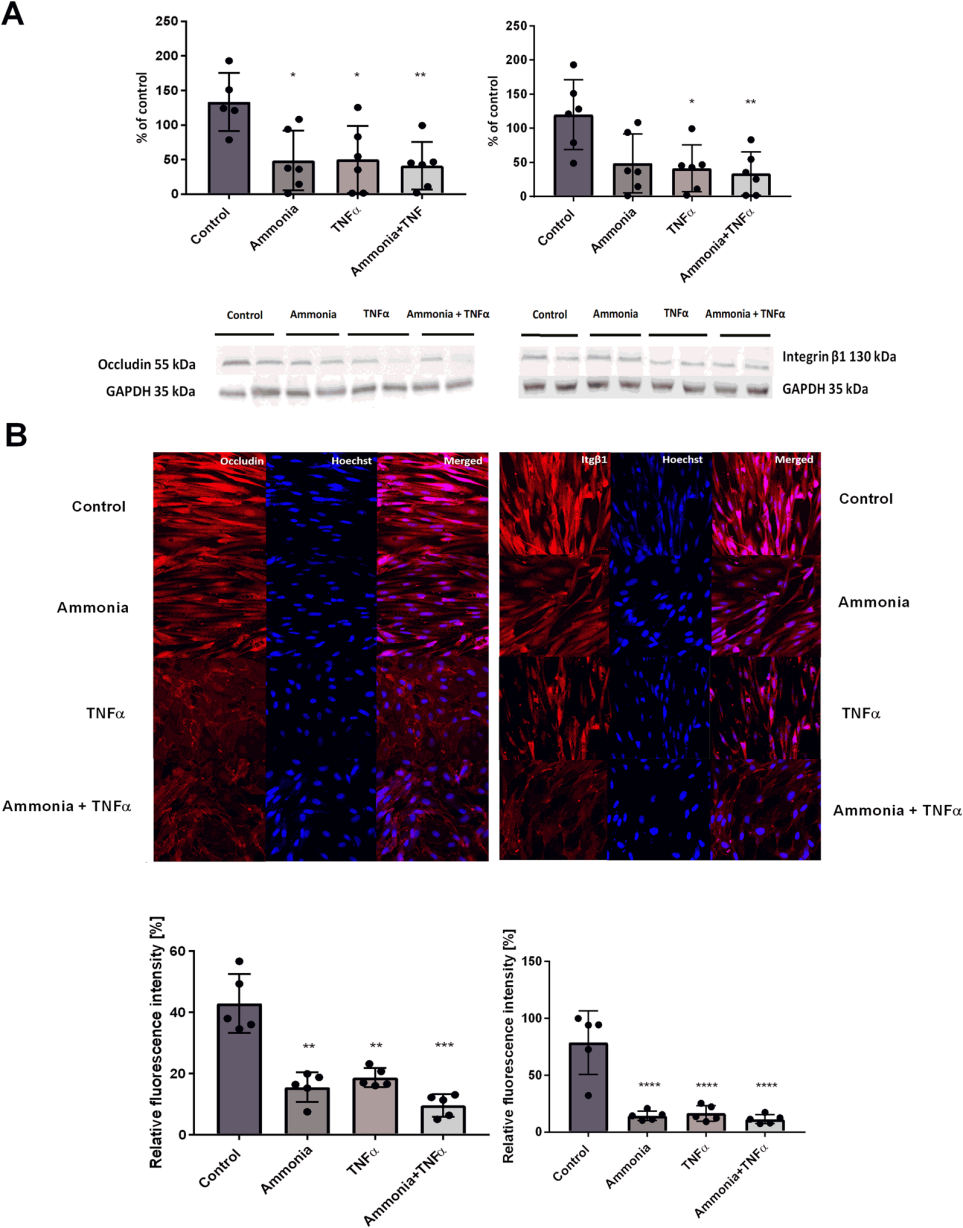
The effect of ammonia and TNFα on occludin and integrin β1 in a rat brain endothelial cell line (RBE4). **A** Protein level of occludin and integrin β1 with representative immunoblots. **B** Confocal microscopy analysis of occludin immunofluorescence (left panel, red), integrin β1 (right panel, red) in RBE4 cells treated or not with ammonia and TNFα. DAPI staining (blue). The results are the mean ± SD; n = 5; *p < 0.05 vs. control, **p < 0.01 vs. control, ***p < 0.01 vs. control, ****p < 0.01 vs. control, one-way ANOVA, and Dunnett’s post hoc test. The results are the mean ± SD. n = 5; *p < 0.05 vs. control, one-way ANOVA, Dunnett’s post hoc test

The RBE4 cells used in further experiments are suited for the analysis of multiple BBB properties. In this experiment, RBE4 cells treated with ammonia, TNFα, or a combination of both factors, demonstrate a decrease in the occludin level with reduced intensity in occludin immunostaining (Fig. 6A, B, left panels). In RBE4 cells treated with TNFα and cells treated with ammonia and TNFα, the protein level of integrin β1 and immunostaining intensity was reduced (Fig. 6A, B, right panels).

**Fig. 7:**
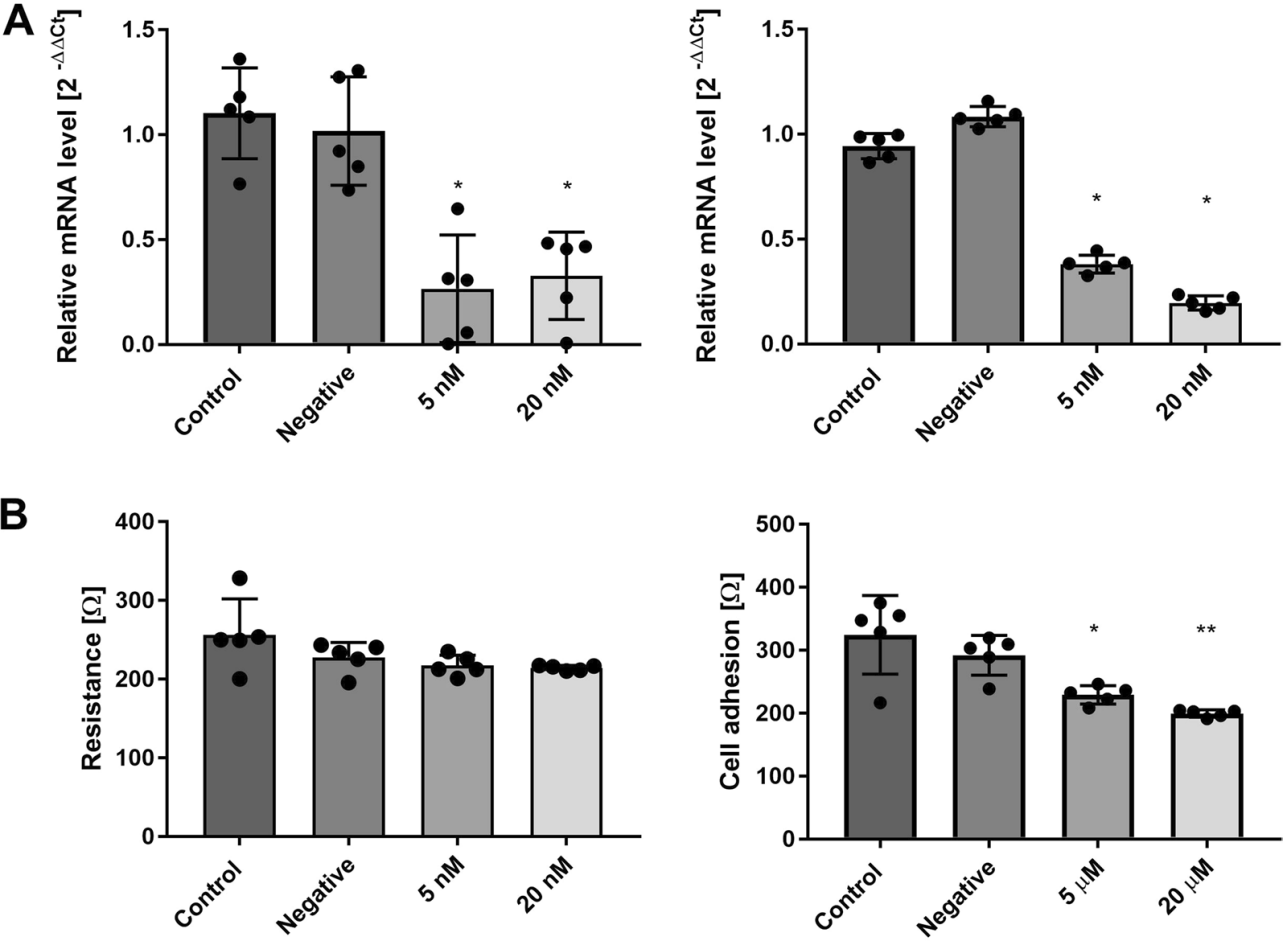
The effect of transfection with miR-122-5p mimic and miR-183-5p mimic on occludin and integrin β1. **A** The effect of transfection with miR 122-5p mimic and miR 183-5p mimic on the mRNA expression level of occludin (left panel) and integrin β1 (right panel), respectively. The results are the mean ± SD. n = 5, *p < 0.05 vs. control, one-way ANOVA, Dunnett’s post hoc test. **B** The effect of transfection with miR-122-5p mimic and miR-183-5p mimic on transendothelial electrical resistance measurement (left panel), and cell adhesion to collagen 1 (right panel). The results are the mean ± SD. n = 5; *p < 0.05 vs. control, **p < 0.01 vs. control, one-way ANOVA, and Dunnett’s post hoc test

In the following experiment, transfection of RBE4 cells with miR-122-5p mimic reduced the expression level of occludin by approximately threefold (Fig. 7A, left panel). Parallelly, RBE4 cell transfection with miR-183-5p mimic significantly decreased the expression of integrin β1 (Fig. 7B, right panel). Since occludin is a key element of TJ proteins [24] and contributes to proper BBB function, we further evaluated the effect of miR-122-5p mimic transfection on the tightness of RBE4 cell monolayer, BBB in vitro model, by transendothelial electrical resistance (TEER) measurement. The unchanged value of resistance of RBE4 cells monolayer was observed (Fig. 7B, left panel), implicating the complexity of TJ protein(s) assembly functioning. In turn, cells transfected with mimic miR-183-5p present a significant reduction of cell adhesion to collagen I (15% and 30% reduction, respectively; Fig. 7B, right panel), interpreted as disturbances in integrin β1-mediated interaction within cellular components of BBB and the extracellular matrix elements [25].

## Discussion

Our study implicates, to our knowledge for the first time, a role of specifically identified miRs in BBB impairment associated with acute HE. First, BBB morphological evaluation by electron microscopy revealed BBB structural alterations with noticeable BBB leakage in the TAA model of ALF with less pronounced changes in the pure hyperammonemia animal model. Second, NGS analysis of plasma from both animal models identified miR-122-5p and miR-183-5p with their targets: occludin and integrin β1 respectively, among target genes related to the BBB structural and functional changes. The identified targets were decreased in TAA rat brain homogenates and microvessels isolated from rat brains in the TAA model, but much less so in simple hyperammonemia. Finally, the verification of selected miRs in cultured endothelial cells treated with critical factors for the pathology of HE, ammonia, and/or TNFα documented at the molecular level miRs’ effects in the endothelium. The study unraveled that miR-185-5p via integrin β1 decline, disturbs integrin β1-mediated interaction within cellular BBB components and the extracellular matrix elements, thus limiting the ability of BBB to execute and control proper biological phenotype.

BBB structural and functional changes were documented in acute, chronic, and acute-upon-chronic disease stages in humans [26–28]. Of note, BBB leakage was also documented in both acute and chronic HE models [15, 29] also in animal models of chronic liver diseases [30–32] However, the complexity of the pathogenic events underlying HE that take place all along the liver - brain axis [32–34] limits the progress in translational studies to prevent BBB dysfunction associated with impaired liver function. The present study illustrates a potentially effective venue towards this end.

We analyze BBB permeability in vivo using two markers: Evans blue (EB), the most commonly used vascular permeability marker which, readily binds to plasma albumin forming a permeability marker suitable for detecting significant BBB leakage. Contrary, low molecular weight (376 Da) dye, SF, enables the detection of relatively more subtle alterations [33]. Using these approaches, we documented that TAA-induced ALF, but not OA, causes structural BBB abnormalities and increases for low molecular weight molecules. Concurrently to the TAA model, limited disturbances related to BBB function in OA rats suggest that an elevated ammonia level alone is not sufficient to cause the BBB disruption in vivo, implicating the contribution of systemic inflammation associated with liver failure. Indeed, ammonia-cytokine synergy in causing brain dysfunction has been repeatedly documented (reviewed in ref. [18]). Human cerebrovascular endothelial cells increase ammonia uptake when exposed to TNFα in vitro [34]. Worth noting, that the expression levels of genes coding enzymes involved in oxidative stress were elevated in cultured astrocytes exposed to both cytokines and ammonia [35]. Down the same valley, chronic hyperammonemia-induced peripheral inflammation with microglia activation and increased proinflammatory cytokines in the hippocampus. Alterations were associated with impaired spatial learning and alleviated by anti-TNFα or sulforaphane treatment [36, 37]. Importantly, the role of inflammatory factors in BBB disruption has been widely documented in other neurological syndromes [38].

Kato and colleagues described swollen astrocytic endfeet, increased number of vacuoles and vesicles in endothelial cells and pericytes, basal lamina enlargement, and intact TJs in post-mortem sections of the brains of ALF patients, thereby documenting BBB structural changes [8]. Additionally, a metabolic study carried out on HE patients revealed ammonia easier entry into the brains of HE patients than in healthy volunteers [39]. A recent study showed that the factors abundant in HE patients’ plasma, enhanced transendothelial leukocyte migration through the in vitro model of BBB, interpreted as elevated permeabilization of the BBB [40].

At the preclinical level, the BBB increased permeability was observed in the hepatotoxic HE model, induced by azoxymethane (AOM), correlating with the progression of liver injury and systemic inflammation [41]. The BBB opening at the symptomatic stage contributed to the neurological decline, as was presented previously [42]. It was documented, that the TAA model leads to cerebral edema as assessed by MRI, though the mechanism has not been elucidated in detail [43]. In the comparative study, the administration of hepatotoxin, TAA increased BBB permeability, measured by EB binding in brain tissue [15]. Complementary, the in vitro experiments using the BBB cellular model in co-culture of brain endothelial cells and astrocytes, documented monolayer increased permeability to AOM but not TAA, indicating direct drug toxicity [15]. Importantly, it was documented that liver injury and resulting inflammation work in concert with the BBB disruption [15]. Our work revealed that enhanced proinflammatory response is noticed in the TAA-injected rats. An enhanced BBB permeability was recently described in the mice model of TAA-induced ALF [15]. Of note, in the bile duct ligation (BDL) rat model of chronic HE, increased BBB permeability to SF and FITC-dextran in multiple brain regions, including the cerebral cortex, were also detected [44]. Regarding HE animal models, in acetaminophen-induced ALF mice increased BBB permeability positively correlates with blood TNFα level, a pleiotropic cytokine and key mediator of inflammation that regulates numerous physiological functions including the development of autoimmunity; the BBB changes in this model were found prevented by anti-TNFα-IgG administration [45]. Moreover, deactivation of the circulating TNFα by TNFα antibody administration increases expression of occludin and zonula occludens1 and decreases BBB permeability for EB day in brains of mice with ALF induced by D-galactosamine and lipopolysaccharide (LPS) administration [46]. Worth noting in this context is that brain endothelial cells do not produce TNFα nor IL-6 in a healthy microenvironment, but rapidly react to challenges (e.g., LPS stimulation), which induces endothelial TNFα and IL-6 production [47].

Less is known about the exact molecular mechanisms underlying BBB permeability increase in HE patients and HE animal models. With the development of bioinformatics tools, the impact of miR in different biological processes control is gradually discovered, however, very limited experimental data documents miRs’ role regarding BBB dysfunction reported in the course of HE.

Increasing evidence points to miR expression changes in body fluids and tissues from both HE patients [11, 48–51] and animal models [52–56] but only a few studies used NGS or other large-scale methods showing whole miRNome changes [57, 58].

In the presented work, we performed NGS profiling of miRs isolated from the plasma of OA and TAA rats, revealing notable differences between experimental groups. We found 35 differently expressed miR in the OA group and 128 in the TAA rat plasma. These analyses allowed us to prove the impact of ammonia on miR expression. Worth noting that the majority of the miRNA upregulated in the OA group (83%) were also increased in TAA indicating an analogous effect of the hyperammonaemia in both models.

The pathomechanism of HE is complex, but increased ammonia concentration in the blood remains the most distinctive event [59, 60]. However, the direct association between HE severity and ammonia level is not certain and has been frequently questioned [61–63]. The direct impact of ammonia on miR expression changes is not fully revealed despite a few reported miRNome dysregulation in clinical studies conducted on patients suffering from acute HE, mostly due to paracetamol over-dose [49, 51, 64–68], or chronic HE: most of the cases were associated with hyperammonaemia [69–71].

Since the impact of relevant changes in altered miR depends on their gene targets, bioinformatic tools serve to select and further investigate miR to predict final functional outcomes [72]. Nevertheless, miR target prediction should be interpreted with caution. Available bioinformatic tools use different algorithms and commercial programs as well as consecutively upgraded databases. Moreover, identified novel sequences added to the databases determine the prediction scores [73–75].

We performed predictive in silico analysis of the gene targets of the differently expressed miR obtained via NGS. We used three different databases: miRDB v5, TargetScan7.1, and TarBase v.8, and selected exclusively overlapped targets as the most certain. Further, we applied a functional prediction method using Gene Ontology (GO) and KEGG Pathways analysis that identified entire biological pathways statistically significantly enriched for selected miR targets [53] to potentiate the overall impact of the analysis, being aware that a single miR regulates hundreds of genes [76].

Our analysis selected miR-122-5p and miR-183-5p from dysregulated miR in a TAA rat plasma and identified occludin and integrin β1 respectively, as their most confirmed targets involved in biological processes associated with the BBB functioning. Noteworthy, we did not obtain output results of our in silico analysis for miR differently expressed in the OA group which is in line with observed unaltered BBB integrity in those animals. These results also indirectly strengthen the hypothesis of the miR-122-5p and miR-183-5p involvement in BBB disturbances described in TAA rats, which we verified in subsequent experiments.

Using the in vitro BBB model, we documented the contribution of selected miR to the reduction of the expression of their targets, the endothelium-located BBB components, occludin, and integrin β1. Consistently we showed that in cultured primary endothelial cells, treatment with ammonia and ammonia with TNFα treatment upregulated miR-122-5p.

The brain microvascular endothelial cells are primary components of the BBB regulating the BBB permeability by junctional proteins’ structures composed of TJ proteins, integrins, annexins, etc., which cooperate with basement membrane components [77]. We used an RBE4 cell line that preserves an endothelial phenotype and presents a structural composition inherent to the in situ BBB, including the presence of TJ and adherence junction proteins [78]. Using this in vitro system, we tested the hypothesis that miR-122-5p affects BBB permeability by regulating the expression of occludin, a main structural protein of TJs presenting a protective effect on different cell barriers [79]. The transfection of RBE4 cells with a miR-122-5p mimic reduced the expression level of occludin by ~ threefold. In our experimental setting, we did not document functionally the impact of occludin on cells’ monolayer tightness. This observation is in line with both in vitro and in vivo studies documenting that occludin controls the passage of macromolecules of ~ 10–70 kDa through mice epithelium without affecting transepithelial resistance [80]. Beyond its structural roles, occludin also plays signaling roles at the BBB; however, molecular details of this process are incompletely understood [81, 82]. Moreover, altered physiological conditions (e.g., ammonia, and cytokines) may differentially affect the organization of occludin isoforms within plasma membrane lipid rafts. Thus, a detailed investigation of protein interactions of particular occludin isoforms will reveal how different signaling and regulatory molecules modulate occludin oligomeric assemblies to promote changes in TJ integrity at the BBB in the setting of ALF. Interestingly in this context, it was also shown that miR-122-5p containing exosomes secreted from LPS-induced neutrophils regulates beyond brain microvascular endothelial cells proliferation and apoptosis, as well as cell monolayer permeability [10].

miR-122 is the most abundant miR in hepatic tissue, constituting the majority of total miR (about 70%) [57]. Reduction of miR-122-5p was observed in the liver of rats receiving low doses of TAA (50 mg/kg of b.w.) for 14 days [83] and also after a short 24 h exposition to a higher (150 mg/kg of b.w.) TAA dose [84]. The authors do not conclude if this phenomenon relies on hepatocyte loss and transfer of miR-122-5p from liver tissue to the blood or if it is a regulatory effect. One of the proposed roles of miR-122-5p for endothelium function is its involvement in the regulation of cationic amino acid transporter 1 (CAT-1) expression [85]. Amino acid l-arginine is transported via CAT-1 and is used as a substrate for endothelial production of nitric oxide. An inverse correlation between plasma levels of miR-122-5p and expression of CAT-1 was reported in a large group of patients with essential hypertension [86]. In the study carried out on TAA rat liver tissue, miR-122-5p reduction was reported to parallel CAT-1 expression upregulation [84]. Indeed, our previous work documented the absence of changes in CAT-1 expression in rats with simple hyperammonemia [37] which is compatible with the unchanged plasma level of miR-122-5p in the OA rats. However, the mechanistic nature of the link between the two events requires further investigation.

Cell-adhesion-related pathways include among others, focal adhesion, integrin signalling, TJ, and actin cytoskeleton regulation, all of the above being linked with miR-183-5p [87, 88]. Bioinformatics databases pointed to a putative binding site of miR-183-5p in the 3′-UTR of integrin 1β. Integrin β1 is a member of the integrin family, which is composed of the major cell surface receptors that mediate adhesion to the extracellular matrix. We validated the negative modulatory role of miR-183-5p in endothelial cell adhesion in the in vitro system, where RBE4 cell transfection was followed by an adhesion assay. We found a significant correlation between miR-183-5p and integrin β1 expression in the brain homogenate samples from TAA rats. Next, we demonstrated that miR-183-5p modulates integrin β1 expression at both the mRNA and protein levels in vitro. Similarly, Dambal et al., found that cell adhesion is the main pathway downregulated in the human prostatic epithelial cell line overexpressing the full miR-183 cluster comprised of miR-96, miR-182, and miR-183-5p [89].

Growing evidence demonstrates that miR-183-5p plays a crucial role in multiple processes of cancer progression by affecting tumor cell interactions [90–92]. These were documented in situ in cervical cancer cell lines transfected with miR-183-5p [93] and human embryonic kidney cells [94]. In this experiment, overexpression of integrin 1β observed in cervical carcinoma samples was negatively correlated with miR-183 level [93]. Another study reported that transfection of HeLa cells with miR-183 decreases cell invasion, but not cell adhesion by down-regulation of integrin 1β [94]. The role of miR-183 in the maintenance of cell integrity in epithelial cells under changing physiological conditions is poorly understood. An increase of miR-183-5p was recently described in mice with intracerebral hemorrhage (ICH) [95], a pathology assessed inter alia with vasogenic edema and BBB breakdown [96]. Administration of miR-183-5p agomir or antagomir into the lateral ventricles reduced neurologic deficits, BBB permeability, brain injury volume, and oxidative damage after ICH [95].

The miR-183-5p was upregulated in BDL-induced liver fibrosis and activated human hepatic stellate cells (LX-2 cell line) [97]. Accordingly, miR-183-5p inhibition alleviated liver fibrosis and downregulated the expression of related fibrotic biomarkers [97]. Additionally, miR-183-5p inhibition reduced LX-2 cell proliferation and promoted apoptosis. The results showed that miR-183-5p might act as a key regulator of liver fibrosis, and miR-183-5p could promote cholestatic liver fibrosis through the TGFβ signaling pathway [97]. Supportively, another study demonstrated upregulation of the miR-183 family in diethylamino ethylamine-induced hepatic fibrosis, suggesting a role in the progression severity of liver fibrosis [98].

In summary, we found that (i) miR-122-5p and miR-185-5p were upregulated in the rat systemic circulation, and that concurrently, occludin and integrin β1, their downstream target genes, were reduced in the endothelium in rat brain homogenate and brain microvessels isolated from TAA treated rats, and that (ii) the above in vivo effects were faithfully reproduced in vitro, in endothelial cells treated with ammonia/TNFα. Whereas miR-122-5p targeting occludin probably does not critically contribute to the BBB permeability increase, the effect of miR-185-5p through integrin β1 decline, presents disturbed integrin β1-mediated interaction within cellular BBB components and the extracellular matrix elements [25], thus limits the ability of BBB to execute proper biological phenotype. Through this study, the molecular mechanism of miR-183-5p as an inhibitory factor affecting endothelial cells at points critical for their BBB has been partially elucidated. The present work indicates that changes in miR, which have straightforward consequences for the expression of endothelial TJ proteins, may be potential indicators of BBB disturbances associated with ALF. As such the changes could be exploited in translational studies to prevent BBB dysfunction occurring due to impaired liver function. The results appear valuable for unraveling further the molecular mechanism of BBB remodeling, likely to be linked to other liver impairment disorders. In terms of scientific perspective, the detection of changes in miR patterns has become a major focus of diagnostic, prognostic, and disease progression, as well as therapy-response markers using a great variety of detection systems in the future.

### Supplementary Information

**Additional file 1: Table S1.** Results of next-generation sequencing of the OA rat plasma. **Table S2.** Results of next-generation sequencing of the TAA rat plasma. **Table S3.** Results of miRs target research in the TAA rat group. **Table S4.** Results of miRs target research in OA rat group. **Figure S1.** miRs tissue specificity, human TissueAtlas miR-183-5p. **Figure S2.** miRs tissue specificity, human TissueAtlas miR-122-5p. **Figure S3.** Immunocytochemical staining of rat brain microvascular endothelial cells with endothelial cell marker. **Figure S4.** Immunocytochemical staining of RBE4 cells with endothelial cell markers.

## Data Availability

The datasets used and/or analysed during this study are available from the corresponding author upon reasonable request.

## Abbreviations

ALF: Acute liver failure
ALT: Alanine aminotransferase
AST: Aspartate aminotransferase
BBB: Blood–brain barrier
HE: Hepatic encephalopathy
ip.: Intraperitoneal
NGS: Next Generation Sequencing
OA: Ammonium acetate/here used to refer to a rat model of hyperammonemia
PBMEC: Primary rat brain microvascular endothelial cells
RBE4: Rat brain endothelial cell line 4
TAA: Thioacetamide//here used to refer to a rat model of ALF
TEER: Transendothelial Electrical Resistance
TEM: Transmission electron microscopy
