# Supplementary material for: miRNA-ome plasma analysis unveils changes in blood–brain barrier integrity associated with acute liver failure in rats
Source: Fluids Barriers CNS. 2023 Dec 8;20:92. doi: 10.1186/s12987-023-00484-7 (PMC10709860; doi:10.1186/s12987-023-00484-7)
Supplement: Supplementary file 1 — Additional file 1: Table S1. Results of next-generation sequencing of the OA rat plasma. Table S2. Results of next-generation sequencing of the TAA rat plasma. Table S3. Results of miRs target research in the TAA rat group. Table S4. Results of miRs target research in OA rat group. Figure S1. miRs tissue specificity, human TissueAtlas miR-183-5p. Figure S2. miRs tissue specificity, human TissueAtlas miR-122-5p. Figure S3. Immunocytochemical staining of rat brain microvascular endothelial cells with endothelial cell marker. Figure S4. Immunocytochemical staining of RBE4 cells with endothelial cell markers. [file 12987_2023_484_MOESM1_ESM.docx]

**Additional file 1:**

**MiRNA-ome plasma analysis unveils changes in blood-brain barrier integrity associated with acute liver failure in rats**

Karolina Orzeł- Gajowik, Krzysztof Milewski, Magdalena Zielińska

E-mail: [mzielinska@imdik.pan.pl](mailto:mzielinska@imdik.pan.pl)

This PDF file includes:

Tabs. 1-4

Figs. 1-3

**Table S1**

**Tab. 1 Results of Next Generation Sequencing of the OA rat plasma**

| **mature_miRNA** | **baseMean** | **log2FoldChange** | **lfcSE** | **stat** | **pvalue** | **padj** | **regulation** | **Sample_Control7_counts_normalized** | **Sample_Control9_counts_normalized** | **Sample_Control10_counts_normalized** | **Sample_Control11_counts_normalized** | **Sample_Control14_counts_normalized** | **Sample_OA1_counts_normalized** | **Sample_OA2_counts_normalized** | **Sample_OA3_counts_normalized** | **Sample_OA4_counts_normalized** | **Sample_OA5_counts_normalized** | **Sample_OA6_counts_normalized** | **Sample_Control7_counts_raw** | **Sample_Control9_counts_raw** | **Sample_Control10_counts_raw** | **Sample_Control11_counts_raw** | **Sample_Control14_counts_raw** | **Sample_OA1_counts_raw** | **Sample_OA2_counts_raw** | **Sample_OA3_counts_raw** | **Sample_OA4_counts_raw** | **Sample_OA5_counts_raw** | **Sample_OA6_counts_raw** |
| --- | --- | --- | --- | --- | --- | --- | --- | --- | --- | --- | --- | --- | --- | --- | --- | --- | --- | --- | --- | --- | --- | --- | --- | --- | --- | --- | --- | --- | --- |
| rno-miR-17-1-3p | 3,48936351 | -4,71364659 | 1,03832682 | -4,53965603 | 5,6346E-06 | 0,0009558 | DOWN | 10,6683082 | 10,7007782 | 10,1932054 | 4,4557165 | 1,50743218 | 0 | 0 | 0 | 0,85755824 | 0 | 0 | 17 | 12 | 14 | 3 | 2 | 0 | 0 | 0 | 1 | 0 | 0 |
| rno-miR-190a-5p | 3,34210736 | -4,65074108 | 1,03644947 | -4,48718556 | 7,217E-06 | 0,0009558 | DOWN | 10,6683082 | 9,80904664 | 9,46511929 | 4,4557165 | 1,50743218 | 0 | 0 | 0 | 0,85755824 | 0 | 0 | 17 | 11 | 13 | 3 | 2 | 0 | 0 | 0 | 1 | 0 | 0 |
| rno-miR-212-5p | 3,34210736 | -4,65074108 | 1,03644947 | -4,48718556 | 7,217E-06 | 0,0009558 | DOWN | 10,6683082 | 9,80904664 | 9,46511929 | 4,4557165 | 1,50743218 | 0 | 0 | 0 | 0,85755824 | 0 | 0 | 17 | 11 | 13 | 3 | 2 | 0 | 0 | 0 | 1 | 0 | 0 |
| rno-miR-296-3p | 3,19485122 | -4,58487154 | 1,03559205 | -4,42729504 | 9,5422E-06 | 0,0009558 | DOWN | 10,6683082 | 8,91731513 | 8,73703319 | 4,4557165 | 1,50743218 | 0 | 0 | 0 | 0,85755824 | 0 | 0 | 17 | 10 | 12 | 3 | 2 | 0 | 0 | 0 | 1 | 0 | 0 |
| rno-miR-148a-5p | 3,80264988 | -4,29330679 | 0,99672594 | -4,30740952 | 1,6518E-05 | 0,00111377 | DOWN | 10,6683082 | 10,7007782 | 10,9212915 | 4,4557165 | 2,26114827 | 0 | 0 | 1,96434793 | 0,85755824 | 0 | 0 | 17 | 12 | 15 | 3 | 3 | 0 | 0 | 1 | 1 | 0 | 0 |
| rno-miR-148b-3p | 3,80264988 | -4,29330679 | 0,99672594 | -4,30740952 | 1,6518E-05 | 0,00111377 | DOWN | 10,6683082 | 10,7007782 | 10,9212915 | 4,4557165 | 2,26114827 | 0 | 0 | 1,96434793 | 0,85755824 | 0 | 0 | 17 | 12 | 15 | 3 | 3 | 0 | 0 | 1 | 1 | 0 | 0 |
| rno-miR-466b-3p | 4,9239564 | -3,12500842 | 0,74186859 | -4,21234763 | 2,5273E-05 | 0,00124593 | DOWN | 11,9234032 | 12,4842412 | 12,3774637 | 7,42619417 | 3,01486435 | 0,85022872 | 1,35776293 | 1,96434793 | 1,71511648 | 1,04989776 | 0 | 19 | 14 | 17 | 5 | 4 | 1 | 1 | 1 | 2 | 1 | 0 |
| rno-miR-370-3p | 2,978763 | -4,49066482 | 1,05758836 | -4,24613677 | 2,1749E-05 | 0,00124593 | DOWN | 10,6683082 | 8,02558362 | 8,73703319 | 2,97047767 | 1,50743218 | 0 | 0 | 0 | 0,85755824 | 0 | 0 | 17 | 9 | 12 | 2 | 2 | 0 | 0 | 0 | 1 | 0 | 0 |
| rno-miR-488-3p | 2,8976965 | -4,45127071 | 1,05919267 | -4,20251275 | 2,6397E-05 | 0,00124593 | DOWN | 10,6683082 | 7,1338521 | 8,73703319 | 2,97047767 | 1,50743218 | 0 | 0 | 0 | 0,85755824 | 0 | 0 | 17 | 8 | 12 | 2 | 2 | 0 | 0 | 0 | 1 | 0 | 0 |
| rno-miR-181d-3p | 2,77445708 | -4,38641412 | 1,0555926 | -4,15540437 | 3,2471E-05 | 0,00139331 | DOWN | 10,0407606 | 7,1338521 | 8,00894709 | 2,97047767 | 1,50743218 | 0 | 0 | 0 | 0,85755824 | 0 | 0 | 16 | 8 | 11 | 2 | 2 | 0 | 0 | 0 | 1 | 0 | 0 |
| rno-miR-1247-3p | 3,97916164 | -3,857521 | 0,94927642 | -4,06364354 | 4,8313E-05 | 0,00175412 | DOWN | 10,6683082 | 11,5925097 | 10,9212915 | 4,4557165 | 2,26114827 | 0 | 0 | 1,96434793 | 0,85755824 | 1,04989776 | 0 | 17 | 13 | 15 | 3 | 3 | 0 | 0 | 1 | 1 | 1 | 0 |
| rno-miR-3547 | 2,62720094 | -4,30696697 | 1,05818732 | -4,07013664 | 4,6986E-05 | 0,00175412 | DOWN | 10,0407606 | 6,24212059 | 7,28086099 | 2,97047767 | 1,50743218 | 0 | 0 | 0 | 0,85755824 | 0 | 0 | 16 | 7 | 10 | 2 | 2 | 0 | 0 | 0 | 1 | 0 | 0 |
| rno-miR-146a-3p | 4,51988211 | -3,26264838 | 0,82144415 | -3,97184444 | 7,1318E-05 | 0,00240445 | DOWN | 11,2958557 | 12,4842412 | 11,6493776 | 5,94095533 | 2,26114827 | 0 | 1,35776293 | 1,96434793 | 1,71511648 | 1,04989776 | 0 | 18 | 14 | 16 | 4 | 3 | 0 | 1 | 1 | 2 | 1 | 0 |
| rno-miR-3594-5p | 4,15964441 | -3,4721404 | 0,87972329 | -3,94685514 | 7,9184E-05 | 0,00249167 | DOWN | 11,2958557 | 11,5925097 | 10,9212915 | 4,4557165 | 2,26114827 | 0 | 1,35776293 | 1,96434793 | 0,85755824 | 1,04989776 | 0 | 18 | 13 | 15 | 3 | 3 | 0 | 1 | 1 | 1 | 1 | 0 |
| rno-miR-135a-5p | 4,59717563 | -3,01313335 | 0,76713651 | -3,92776685 | 8,5738E-05 | 0,00252928 | DOWN | 11,2958557 | 12,4842412 | 11,6493776 | 5,94095533 | 2,26114827 | 0,85022872 | 1,35776293 | 1,96434793 | 1,71511648 | 1,04989776 | 0 | 18 | 14 | 16 | 4 | 3 | 1 | 1 | 1 | 2 | 1 | 0 |
| rno-miR-3075 | 4,30379389 | -3,18881681 | 0,83269993 | -3,82949089 | 0,00012841 | 0,00356523 | DOWN | 11,2958557 | 11,5925097 | 11,6493776 | 4,4557165 | 2,26114827 | 0 | 1,35776293 | 1,96434793 | 1,71511648 | 1,04989776 | 0 | 18 | 13 | 16 | 3 | 3 | 0 | 1 | 1 | 2 | 1 | 0 |
| rno-miR-935 | 2,17055564 | -4,02645643 | 1,1006494 | -3,65825523 | 0,00025394 | 0,00630836 | DOWN | 10,0407606 | 2,67519454 | 5,82468879 | 2,97047767 | 1,50743218 | 0 | 0 | 0 | 0,85755824 | 0 | 0 | 16 | 3 | 8 | 2 | 2 | 0 | 0 | 0 | 1 | 0 | 0 |
| rno-miR-296-5p | 1,98112657 | -3,88854268 | 1,10238618 | -3,52738699 | 0,00041968 | 0,00977864 | DOWN | 9,41321308 | 2,67519454 | 4,3685166 | 2,97047767 | 1,50743218 | 0 | 0 | 0 | 0,85755824 | 0 | 0 | 15 | 3 | 6 | 2 | 2 | 0 | 0 | 0 | 1 | 0 | 0 |
| rno-miR-410-5p | 1,91493693 | -3,83659102 | 1,109837 | -3,45689594 | 0,00054644 | 0,01172353 | DOWN | 9,41321308 | 2,67519454 | 3,6404305 | 2,97047767 | 1,50743218 | 0 | 0 | 0 | 0,85755824 | 0 | 0 | 15 | 3 | 5 | 2 | 2 | 0 | 0 | 0 | 1 | 0 | 0 |
| rno-miR-1298 | 1,79169751 | -3,73402675 | 1,11509072 | -3,34863045 | 0,00081212 | 0,0159717 | DOWN | 8,78566554 | 2,67519454 | 2,9123444 | 2,97047767 | 1,50743218 | 0 | 0 | 0 | 0,85755824 | 0 | 0 | 14 | 3 | 4 | 2 | 2 | 0 | 0 | 0 | 1 | 0 | 0 |
| rno-miR-376b-3p | 5,6857635 | -2,13723271 | 0,70800213 | -3,0186812 | 0,00253878 | 0,0386549 | DOWN | 11,9234032 | 13,3759727 | 12,3774637 | 8,911433 | 3,01486435 | 0,85022872 | 1,35776293 | 1,96434793 | 7,71802418 | 1,04989776 | 0 | 19 | 15 | 17 | 6 | 4 | 1 | 1 | 1 | 9 | 1 | 0 |
| rno-miR-802-3p | 6,97618532 | -1,94503716 | 0,65828649 | -2,95469708 | 0,00312976 | 0,04476508 | DOWN | 12,5509508 | 14,2677042 | 13,8336359 | 11,8819107 | 6,7834448 | 0,85022872 | 1,35776293 | 1,96434793 | 11,1482571 | 2,09979551 | 0 | 20 | 16 | 19 | 8 | 9 | 1 | 1 | 1 | 13 | 2 | 0 |
| rno-miR-222-5p | 1,57592171 | -3,5346721 | 1,20412386 | -2,93547218 | 0,0033304 | 0,04491289 | DOWN | 8,78566554 | 1,78346303 | 2,1842583 | 2,97047767 | 0,75371609 | 0 | 0 | 0 | 0,85755824 | 0 | 0 | 14 | 2 | 3 | 2 | 1 | 0 | 0 | 0 | 1 | 0 | 0 |
| rno-let-7c-5p | 2278374,41 | 1,36698372 | 0,30965852 | 4,41448766 | 1,0125E-05 | 0,0009558 | UP | 1217721,48 | 1703690,51 | 1022317,34 | 995369,935 | 1180827,4 | 5044103,46 | 2280235,21 | 1730790,89 | 1958061,02 | 3924443,27 | 4004558,03 | 1940445 | 1910542 | 1404116 | 670175 | 1566674 | 5932643 | 1679406 | 881102 | 2283298 | 3737929 | 3953984 |
| rno-miR-429 | 63631,2412 | 0,27323572 | 0,07378026 | 3,70337175 | 0,00021275 | 0,00557885 | UP | 61158,2729 | 50055,565 | 64129,8236 | 54298,8465 | 56014,6723 | 68163,6867 | 68968,9259 | 71537,6227 | 60371,2427 | 70798,8053 | 74446,1896 | 97456 | 56133 | 88080 | 36559 | 74318 | 80171 | 50796 | 36418 | 70399 | 67434 | 73506 |
| rno-miR-369-5p | 75958,6901 | 0,27794314 | 0,0790095 | 3,51784481 | 0,00043507 | 0,00977864 | UP | 68616,0479 | 60142,8319 | 73924,0378 | 74319,866 | 63355,1133 | 90433,7275 | 79919,2839 | 75780,6143 | 72889,0203 | 87664,3629 | 88500,6854 | 109340 | 67445 | 101532 | 50039 | 84057 | 106364 | 58861 | 38578 | 84996 | 83498 | 87383 |
| rno-miR-149-5p | 71068,9644 | 0,27316923 | 0,08146922 | 3,35303609 | 0,0007993 | 0,0159717 | UP | 67397,9781 | 55931,184 | 71834,4307 | 64781,6622 | 59119,2289 | 74031,9653 | 74514,0297 | 75503,6412 | 68006,0837 | 83456,3727 | 87182,032 | 107399 | 62722 | 98662 | 43617 | 78437 | 87073 | 54880 | 38437 | 79302 | 79490 | 86081 |
| rno-miR-16-5p | 66722,5111 | 0,21821198 | 0,06562609 | 3,32507975 | 0,00088393 | 0,01668865 | UP | 65187,1281 | 55316,7809 | 64523,7182 | 63097,4013 | 58200,4489 | 68648,317 | 73036,7836 | 71853,8828 | 63195,182 | 71736,364 | 79151,6149 | 103876 | 62033 | 88621 | 42483 | 77218 | 80741 | 53792 | 36579 | 73692 | 68327 | 78152 |
| rno-miR-127-3p | 177009,891 | 0,20397729 | 0,06368004 | 3,20315898 | 0,00135929 | 0,02467633 | UP | 171419,631 | 143484,059 | 170431,85 | 163954,03 | 168059,845 | 191589,689 | 175038,724 | 170805,945 | 194715,459 | 203916,392 | 193693,174 | 273158 | 160905 | 234082 | 110389 | 222975 | 225339 | 128917 | 86953 | 227058 | 194225 | 191247 |
| rno-miR-126a-3p | 52861,35 | 0,21081668 | 0,06679654 | 3,15610176 | 0,00159893 | 0,02795168 | UP | 50867,1208 | 43549,4919 | 50345,6976 | 49142,0973 | 49504,8264 | 53867,941 | 54745,0014 | 59765,2856 | 49685,2094 | 57100,7893 | 62901,3889 | 81057 | 48837 | 69148 | 33087 | 65681 | 63357 | 40320 | 30425 | 57938 | 54387 | 62107 |
| rno-let-7f-5p | 81767,8246 | 0,2694388 | 0,08776 | 3,07017779 | 0,00213931 | 0,03606272 | UP | 71312,6196 | 66034,502 | 79047,5797 | 81144,5384 | 70128,7598 | 97180,2924 | 81096,4644 | 80777,9154 | 76843,2214 | 101136,651 | 94743,527 | 113637 | 74052 | 108569 | 54634 | 93044 | 114299 | 59728 | 41122 | 89607 | 96330 | 93547 |
| rno-miR-25-3p | 170248,698 | 0,20869083 | 0,0683592 | 3,0528563 | 0,00226674 | 0,03689322 | UP | 160690,45 | 137150,09 | 170299,339 | 158002,678 | 158496,695 | 185932,267 | 160824,304 | 168873,027 | 193603,206 | 188150,077 | 190713,543 | 256061 | 153802 | 233900 | 106382 | 210287 | 218685 | 118448 | 85969 | 225761 | 179208 | 188305 |
| rno-miR-34b-3p | 4118,59904 | 0,1269878 | 0,04194718 | 3,02732611 | 0,00246728 | 0,0386549 | UP | 3970,49328 | 3825,52819 | 4129,70436 | 4068,06916 | 3618,59094 | 4269,84863 | 4103,15958 | 4384,42457 | 4295,50923 | 4274,13377 | 4365,1277 | 6327 | 4290 | 5672 | 2739 | 4801 | 5022 | 3022 | 2232 | 5009 | 4071 | 4310 |
| rno-miR-181a-5p | 139556,859 | 0,27545661 | 0,09193276 | 2,99628351 | 0,00273292 | 0,0403106 | UP | 127272,916 | 108614,682 | 155982,254 | 124446,677 | 109640,818 | 155720,24 | 139587,534 | 140197,476 | 153569,815 | 161117,31 | 158975,723 | 202810 | 121802 | 214236 | 83789 | 145467 | 183151 | 102807 | 71371 | 179078 | 153460 | 156968 |
| rno-miR-146a-5p | 163840,252 | 0,18678207 | 0,06362041 | 2,93588311 | 0,003326 | 0,04491289 | UP | 147017,444 | 136548,171 | 164004,306 | 156251,581 | 157954,02 | 173100,616 | 153211,327 | 167334,942 | 181003,961 | 184727,41 | 181088,994 | 234273 | 153127 | 225254 | 105203 | 209567 | 203593 | 112841 | 85186 | 211069 | 175948 | 178802 |

**Table S2**

**Tab. 2 Results of Next Generation Sequencing of the TAA rat plasma**

| **mature_miRNA** | **baseMean** | **log2FoldChange** | **lfcSE** | **stat** | **pvalue** | **padj** | **regulation** | **Sample_Control7_counts_normalized** | **Sample_Control9_counts_normalized** | **Sample_Control10_counts_normalized** | **Sample_Control11_counts_normalized** | **Sample_Control14_counts_normalized** | **Sample_TAA1_counts_normalized** | **Sample_TAA2_counts_normalized** | **Sample_TAA6_counts_normalized** | **Sample_TAA8_counts_normalized** | **Sample_TAA9_counts_normalized** | **Sample_TAA11_counts_normalized** | **Sample_Control7_counts_raw** | **Sample_Control9_counts_raw** | **Sample_Control10_counts_raw** | **Sample_Control11_counts_raw** | **Sample_Control14_counts_raw** | **Sample_TAA1_counts_raw** | **Sample_TAA2_counts_raw** |
| --- | --- | --- | --- | --- | --- | --- | --- | --- | --- | --- | --- | --- | --- | --- | --- | --- | --- | --- | --- | --- | --- | --- | --- | --- | --- |
| rno-let-7c-5p | 7795650,8 | 3,848604607 | 0,21536715 | 17,8699703 | 2,021E-71 | 9,8424E-69 | UP | 960912,147 | 1290252,3 | 775535,351 | 752913,55 | 909555,907 | 17098471,2 | 8834584,56 | 12071596,7 | 16813878,8 | 15691985 | 10552473,3 | 1940445 | 1910542 | 1404116 | 670175 | 1566674 | 10323516 | 7341543 |
| rno-miR-218a-2-3p | 1,035598072 | 3,34224733 | 1,23888023 | 2,69779698 | 0,00698 | 0,02880729 | UP | 0,49520195 | 0 | 0 | 0 | 0 | 1,65626432 | 2,40673781 | 0 | 1,42758097 | 1,87155784 | 3,5342359 | 1 | 0 | 0 | 0 | 0 | 1 | 2 |
| rno-miR-10b-3p | 1,243527904 | 2,931293416 | 1,1703638 | 2,50460021 | 0,01225899 | 0,04738196 | UP | 0,49520195 | 0 | 0 | 1,12345813 | 0 | 1,65626432 | 2,40673781 | 1,16377002 | 1,42758097 | 1,87155784 | 3,5342359 | 1 | 0 | 0 | 1 | 0 | 1 | 2 |
| rno-miR-148b-5p | 1,243527904 | 2,931293416 | 1,1703638 | 2,50460021 | 0,01225899 | 0,04738196 | UP | 0,49520195 | 0 | 0 | 1,12345813 | 0 | 1,65626432 | 2,40673781 | 1,16377002 | 1,42758097 | 1,87155784 | 3,5342359 | 1 | 0 | 0 | 1 | 0 | 1 | 2 |
| rno-miR-155-3p | 1,243527904 | 2,931293416 | 1,1703638 | 2,50460021 | 0,01225899 | 0,04738196 | UP | 0,49520195 | 0 | 0 | 1,12345813 | 0 | 1,65626432 | 2,40673781 | 1,16377002 | 1,42758097 | 1,87155784 | 3,5342359 | 1 | 0 | 0 | 1 | 0 | 1 | 2 |
| rno-miR-19a-3p | 1,243527904 | 2,931293416 | 1,1703638 | 2,50460021 | 0,01225899 | 0,04738196 | UP | 0,49520195 | 0 | 0 | 1,12345813 | 0 | 1,65626432 | 2,40673781 | 1,16377002 | 1,42758097 | 1,87155784 | 3,5342359 | 1 | 0 | 0 | 1 | 0 | 1 | 2 |
| rno-miR-206-3p | 1830539,382 | 2,270572448 | 0,24263767 | 9,35787263 | 8,1358E-21 | 9,9054E-19 | UP | 889489,17 | 466882,7768 | 488930,224 | 569196,689 | 550952,009 | 3676785,89 | 1748271,19 | 2613591,22 | 3561072,17 | 3283416,17 | 2287345,69 | 1796215 | 691337 | 885214 | 506647 | 948993 | 2219927 | 1452814 |
| rno-miR-1-3p | 455424,6042 | 1,130569533 | 0,19919542 | 5,67568037 | 1,3814E-08 | 2,4916E-07 | UP | 348025,454 | 261811,7954 | 244948,953 | 218308,136 | 307988,509 | 582420,38 | 639637,504 | 442780,743 | 766165,575 | 780110,227 | 417473,369 | 702795 | 387678 | 443483 | 194318 | 530498 | 351647 | 531539 |
| rno-miR-199a-3p | 549201,2189 | 1,065239084 | 0,29189509 | 3,64939026 | 0,00026286 | 0,00150605 | UP | 604824,31 | 263437,9975 | 272808,477 | 233820,846 | 345750,191 | 816563,154 | 667444,953 | 594845,916 | 890387,96 | 846921,099 | 504408,504 | 1221369 | 390086 | 493923 | 208126 | 595541 | 493015 | 554647 |
| rno-miR-23a-3p | 387433,376 | 0,90298829 | 0,14117533 | 6,39621874 | 1,5927E-10 | 4,8478E-09 | UP | 324665,293 | 246512,1234 | 239242,832 | 213715,439 | 289627,564 | 559385,056 | 425005,83 | 363156,762 | 617427,341 | 600886,105 | 382142,791 | 655622 | 365023 | 433152 | 190230 | 498872 | 337739 | 353180 |
| rno-let-7g-5p | 352439,3373 | 0,846791046 | 0,13886621 | 6,09789146 | 1,0748E-09 | 2,6171E-08 | UP | 321659,417 | 239795,2601 | 234984,92 | 188669,064 | 242431,703 | 446139,639 | 419358,42 | 343312,156 | 585725,051 | 508921,495 | 345835,586 | 649552 | 355077 | 425443 | 167936 | 417579 | 269365 | 348487 |
| rno-miR-451-5p | 640943,8507 | 0,829700067 | 0,19124528 | 4,33840806 | 1,4352E-05 | 0,00012943 | UP | 607325,08 | 363433,898 | 356952,634 | 470335,745 | 452471,369 | 1035990,02 | 688227,134 | 673486,511 | 936894,265 | 862047,029 | 603218,671 | 1226419 | 538155 | 646267 | 418650 | 779364 | 625498 | 571917 |
| rno-miR-99a-5p | 308401,8335 | 0,801884755 | 0,13339547 | 6,01133432 | 1,84E-09 | 4,2671E-08 | UP | 320394,176 | 192578,6693 | 229115,862 | 179112,929 | 175934,963 | 415578,25 | 388881,899 | 340243,294 | 448658,719 | 369155,427 | 332765,981 | 646997 | 285161 | 414817 | 159430 | 303041 | 250913 | 323161 |
| rno-miR-149-5p | 65611,79765 | 0,701116713 | 0,07666554 | 9,14513552 | 5,9555E-20 | 5,8007E-18 | UP | 53184,1942 | 42358,24429 | 54493,9797 | 49001,8731 | 45537,7677 | 75277,2134 | 83547,4963 | 78023,7971 | 86302,9799 | 80617,3542 | 73384,8742 | 107399 | 62722 | 98662 | 43617 | 78437 | 45450 | 69428 |
| rno-let-7b-5p | 273224,8329 | 0,689212782 | 0,12694465 | 5,42923846 | 5,6595E-08 | 8,3521E-07 | UP | 271907,467 | 182310,9045 | 226700,523 | 178553,447 | 164575,049 | 404845,657 | 357300,685 | 283526,962 | 358309,975 | 294218,251 | 283224,24 | 549084 | 269957 | 410444 | 158932 | 283474 | 244433 | 296917 |
| rno-miR-369-5p | 68769,68024 | 0,663499669 | 0,06982765 | 9,50196219 | 2,0597E-21 | 3,3436E-19 | UP | 54145,3812 | 45547,84264 | 56079,1667 | 56216,7212 | 48800,5423 | 81580,9554 | 87298,3972 | 82055,0965 | 86447,1655 | 81927,4446 | 76367,7693 | 109340 | 67445 | 101532 | 50039 | 84057 | 49256 | 72545 |
| rno-miR-16-5p | 61886,9254 | 0,662055284 | 0,06909154 | 9,58229145 | 9,4915E-22 | 2,3112E-19 | UP | 51439,5977 | 41892,93976 | 48948,0345 | 47727,8716 | 44830,0591 | 74235,4231 | 71121,509 | 76363,0973 | 82060,2092 | 73443,6729 | 68693,7651 | 103876 | 62033 | 88621 | 42483 | 77218 | 44821 | 59102 |
| rno-miR-429 | 57295,05228 | 0,645601846 | 0,09049824 | 7,13386042 | 9,7592E-13 | 5,2808E-11 | UP | 48260,4012 | 37908,47432 | 48649,224 | 41072,5056 | 43146,4209 | 66507,2938 | 59734,0291 | 66729,4091 | 82035,9403 | 72444,261 | 63757,6156 | 97456 | 56133 | 88080 | 36559 | 74318 | 40155 | 49639 |
| rno-let-7f-5p | 72182,95129 | 0,600290098 | 0,07180806 | 8,35964805 | 6,2906E-17 | 5,1058E-15 | UP | 56273,2639 | 50009,76859 | 59965,9128 | 61379,0113 | 54018,0789 | 81966,865 | 95521,0169 | 84475,7381 | 87136,6871 | 85505,8632 | 77760,2583 | 113637 | 74052 | 108569 | 54634 | 93044 | 49489 | 79378 |
| rno-miR-30d-5p | 53744,03353 | 0,570231355 | 0,07074135 | 8,06079214 | 7,5802E-16 | 5,2736E-14 | UP | 46668,3269 | 37907,79899 | 46878,4541 | 40912,9746 | 40153,609 | 65863,007 | 58210,564 | 66550,1885 | 62803,5695 | 61521,8495 | 63714,0267 | 94241 | 56132 | 84874 | 36417 | 69163 | 39766 | 48373 |
| rno-miR-191a-5p | 226265,6702 | 0,56860059 | 0,12996516 | 4,37502306 | 1,2142E-05 | 0,00011157 | UP | 186936,755 | 175500,1699 | 211873,777 | 170722,944 | 150355,274 | 328667,436 | 318554,613 | 251191,612 | 235610,818 | 281759,29 | 177749,682 | 377496 | 259872 | 383600 | 151962 | 258981 | 198439 | 264719 |
| rno-miR-26a-5p | 240200,2199 | 0,552355748 | 0,13938393 | 3,9628368 | 7,4064E-05 | 0,00050802 | UP | 226551,425 | 180552,337 | 222016,765 | 172895,712 | 155378,321 | 362094,162 | 346889,137 | 256895,249 | 239886,423 | 284074,407 | 194968,48 | 457493 | 267353 | 401964 | 153896 | 267633 | 218621 | 288265 |
| rno-miR-126a-3p | 46072,82661 | 0,515740034 | 0,07158432 | 7,20465045 | 5,8193E-13 | 3,5425E-11 | UP | 40139,5844 | 32981,24384 | 38192,5129 | 37171,859 | 38132,082 | 58045,4394 | 52262,3115 | 56505,6895 | 54525,0275 | 52109,7851 | 46735,5575 | 81057 | 48837 | 69148 | 33087 | 65681 | 35046 | 43430 |
| rno-miR-122-5p | 48008,63649 | 0,503694483 | 0,07968873 | 6,3207743 | 2,6026E-10 | 7,2746E-09 | UP | 42775,5444 | 33508,67901 | 41684,8953 | 39064,886 | 38451,3927 | 61720,6899 | 53161,2281 | 59232,4027 | 56103,9321 | 53925,1962 | 48466,155 | 86380 | 49618 | 75471 | 34772 | 66231 | 37265 | 44177 |
| rno-miR-10a-5p | 51217,31259 | 0,493221075 | 0,07432818 | 6,63572097 | 3,2292E-11 | 1,3105E-09 | UP | 46368,2345 | 36994,7486 | 46629,3532 |  | 38976,2234 | 62164,5688 | 56801,4191 | 62534,0182 | 59844,1942 | 54960,1677 | 57579,7713 | 93635 | 54780 | 84423 | 36083 | 67135 | 37533 | 47202 |
| rno-miR-100-5p | 44472,82853 | 0,467262395 | 0,07129604 | 6,55383375 | 5,6078E-11 | 2,1008E-09 | UP | 39912,2867 | 31398,93833 | 38150,5358 | 36557,3274 | 37957,332 | 55145,3206 | 51191,3132 | 51230,32 | 51308,6876 | 50221,3832 | 46127,6689 | 80598 | 46494 | 69072 | 32540 | 65380 | 33295 | 42540 |
| rno-miR-22-3p | 76491,08891 | 0,46412981 | 0,09405848 | 4,93448132 | 8,0364E-07 | 1,0578E-05 | UP | 78533,0868 | 54774,91913 | 64249,784 | 64707,8177 | 54599,8049 | 82043,0532 | 95557,118 | 85279,9032 | 94485,874 | 88393,677 | 78776,9401 | 158588 | 81108 | 116325 | 57597 | 94046 | 49535 | 79408 |
| rno-miR-127-3p | 150179,9396 | 0,447143823 | 0,11298852 | 3,95742705 | 7,5761E-05 | 0,00051244 | UP | 135268,374 | 108664,4765 | 129290,505 | 124017,419 | 129451,455 | 176054,272 | 169836,267 | 131256,965 | 206321,139 | 204336,685 | 137481,777 | 273158 | 160905 | 234082 | 110389 | 222975 | 106296 | 141134 |
| rno-miR-341 | 37832,22657 | 0,446665057 | 0,08124849 | 5,4975181 | 3,8517E-08 | 6,051E-07 | UP | 36415,1706 | 28746,90514 | 33440,2658 | 29894,0973 | 29402,7085 | 41081,9802 | 39637,7684 | 43328,3216 | 48129,4648 | 46566,2307 | 39511,5793 | 73536 | 42567 | 60544 | 26609 | 50645 | 24804 | 32939 |
| rno-miR-449a-5p | 1109,268485 | 0,420093107 | 0,06553992 | 6,40972864 | 1,4578E-10 | 4,733E-09 | UP | 979,014254 | 895,4917241 | 977,071721 | 878,544255 | 945,740193 | 1273,66726 | 1262,33398 | 1123,03807 | 1319,08481 | 1283,88868 | 1264,07837 | 1977 | 1326 | 1769 | 782 | 1629 | 769 | 1049 |
| rno-miR-455-3p | 1096,953644 | 0,418421331 | 0,06447341 | 6,48982812 | 8,5934E-11 | 2,9893E-09 | UP | 968,615013 | 892,1150585 | 950,007552 | 872,926964 | 944,579064 | 1262,07341 | 1262,33398 | 1113,72791 | 1307,66417 | 1238,97129 | 1253,47567 | 1956 | 1321 | 1720 | 777 | 1627 | 762 | 1049 |
| rno-miR-3559-3p | 1139,430845 | 0,417755645 | 0,06208691 | 6,72856248 | 1,7135E-11 | 8,3446E-10 | UP | 1014,66879 | 913,7257185 | 998,06026 | 903,260334 | 978,251827 | 1320,04266 | 1287,60473 | 1204,50197 | 1340,49853 | 1291,37491 | 1281,74955 | 2049 | 1353 | 1807 | 804 | 1685 | 797 | 1070 |
| rno-miR-130a-3p | 1072,408963 | 0,411086505 | 0,06872395 | 5,98170677 | 2,2081E-09 | 4,888E-08 | UP | 962,67259 | 868,4783991 | 946,141242 | 830,235556 | 929,484377 | 1219,01054 | 1217,80933 | 1082,30612 | 1290,5332 | 1235,22818 | 1214,59907 | 1944 | 1286 | 1713 | 739 | 1601 | 736 | 1012 |
| rno-miR-214-3p | 1169,441045 | 0,405214058 | 0,06060487 | 6,68616309 | 2,291E-11 | 1,0143E-09 | UP | 1027,04884 | 973,8303667 | 1039,48501 | 932,470245 | 988,12143 | 1336,60531 | 1287,60473 | 1224,28606 | 1350,4916 | 1355,00788 | 1348,90004 | 2074 | 1442 | 1882 | 830 | 1702 | 807 | 1070 |
| rno-miR-99b-3p | 990,8404484 | 0,404066436 | 0,06397771 | 6,31573751 | 2,6888E-10 | 7,2746E-09 | UP | 882,945076 | 800,9450866 | 823,523989 | 810,013309 | 888,844835 | 1146,13491 | 1131,16677 | 1021,79008 | 1113,51316 | 1143,52184 | 1136,84588 | 1783 | 1186 | 1491 | 721 | 1531 | 692 | 940 |
| rno-miR-24-3p | 79474,72276 | 0,397949582 | 0,10276807 | 3,87230772 | 0,00010781 | 0,00070004 | UP | 86851,9843 | 59462,40635 | 66551,343 | 71078,9488 | 54747,2684 | 85613,959 | 96344,1213 | 85283,3945 | 94618,639 | 94259,1393 | 79410,7465 | 175387 | 88049 | 120492 | 63268 | 94300 | 51691 | 80062 |
| rno-miR-378a-3p | 205831,925 | 0,397099092 | 0,13109495 | 3,02909524 | 0,00245287 | 0,01106064 | UP | 184289,901 | 171302,2992 | 202703,443 | 170157,844 | 149042,617 | 265527,327 | 282736,338 | 170768,121 | 231707,812 | 268911,046 | 167004,427 | 372151 | 253656 | 366997 | 151459 | 256720 | 160317 | 234954 |
| rno-miR-380-3p | 948,9227693 | 0,396632531 | 0,06647802 | 5,96637144 | 2,4259E-09 | 5,1365E-08 | UP | 854,223363 | 752,9964347 | 799,7738 | 780,803398 | 852,849812 | 1113,00962 | 1015,64336 | 999,678446 | 1050,69959 | 1100,47601 | 1117,99662 | 1725 | 1115 | 1448 | 695 | 1469 | 672 | 844 |
| rno-miR-26b-5p | 35901,0481 | 0,395658702 | 0,08516537 | 4,64576998 | 3,3881E-06 | 4,0244E-05 | UP | 34629,4723 | 27015,35101 | 32819,447 | 29325,6275 | 29350,4576 | 37701,5447 | 38850,7651 | 37421,025 | 46621,9393 | 44213,6825 | 36962,2171 | 69930 | 40003 | 59420 | 26103 | 50555 | 22763 | 32285 |
| rno-miR-466c-5p | 1014,420612 | 0,389882628 | 0,06741701 | 5,78314941 | 7,3315E-09 | 1,4282E-07 | UP | 921,570828 | 804,9970854 | 894,774555 | 812,260226 | 898,133873 | 1156,0725 | 1156,43752 | 1029,93647 | 1153,48542 | 1152,87963 | 1178,07863 | 1861 | 1192 | 1620 | 723 | 1547 | 698 | 961 |
| rno-miR-329-3p | 1055,770874 | 0,38607746 | 0,0670034 | 5,76205737 | 8,3095E-09 | 1,5564E-07 | UP | 962,177388 | 863,0757341 | 936,751633 | 822,371349 | 929,484377 | 1184,22899 | 1204,57227 | 1077,65104 | 1193,45769 | 1233,35662 | 1206,35252 | 1943 | 1278 | 1696 | 732 | 1601 | 715 | 1001 |
| rno-miR-1b | 158869,717 | 0,385947941 | 0,10984436 | 3,51358891 | 0,0004421 | 0,00240525 | UP | 154880,847 | 113668,695 | 147513,527 | 130067,241 | 134363,614 | 181566,32 | 174739,995 | 142137,051 | 211643,161 | 208034,884 | 148951,55 | 312763 | 168315 | 267075 | 115774 | 231436 | 109624 | 145209 |
| rno-miR-337-5p | 970,5362472 | 0,384599478 | 0,06099862 | 6,30505217 | 2,881E-10 | 7,3844E-09 | UP | 865,117806 | 798,9190872 | 820,210009 | 806,642935 | 863,880545 | 1124,60347 | 1073,40506 | 1017,135 | 1082,10637 | 1102,34757 | 1121,53086 | 1747 | 1183 | 1485 | 718 | 1488 | 679 | 892 |
| rno-miR-379-3p | 1026,041011 | 0,38338534 | 0,06849803 | 5,59702716 | 2,1806E-08 | 3,6619E-07 | UP | 935,436483 | 823,2310797 | 918,524744 | 813,383684 | 902,778392 | 1157,72876 | 1181,70826 | 1032,26401 | 1177,7543 | 1158,49431 | 1185,14711 | 1889 | 1219 | 1663 | 724 | 1555 | 699 | 982 |
| rno-miR-497-5p | 1035,280284 | 0,37836791 | 0,07073363 | 5,34919381 | 8,8347E-08 | 1,2654E-06 | UP | 962,177388 | 827,2830785 | 920,734064 | 814,507142 | 917,292514 | 1175,94767 | 1182,91163 | 1035,75532 | 1177,7543 | 1169,72365 | 1203,99636 | 1943 | 1225 | 1667 | 725 | 1580 | 710 | 983 |
| rno-miR-192-5p | 39422,21013 | 0,37756848 | 0,08262628 | 4,56959287 | 4,8867E-06 | 5,5345E-05 | UP | 39718,1676 | 29029,19439 | 34389,1687 | 33762,1636 | 32556,9175 | 42551,0867 | 41314,0612 | 45418,4525 | 48573,4424 | 46586,8179 | 39744,8389 | 80206 | 42985 | 62262 | 30052 | 56078 | 25691 | 34332 |
| rno-miR-93-5p | 41512,80594 | 0,37476852 | 0,07869308 | 4,76240756 | 1,913E-06 | 2,329E-05 | UP | 39790,9622 | 29675,48819 | 36432,2373 | 34897,9798 | 37855,7332 | 46907,0618 | 45098,6565 | 47680,8214 | 49237,2676 | 47893,1652 | 41171,4921 | 80353 | 43942 | 65961 | 31063 | 65205 | 28321 | 37477 |
| rno-miR-25-3p | 139675,6181 | 0,373399967 | 0,10710496 | 3,48629961 | 0,00048975 | 0,00255116 | UP | 126801,906 | 103867,5853 | 129189,981 | 119515,722 | 122085,247 | 157802,239 | 166392,225 | 124660,717 | 188963,182 | 172937,56 | 124215,433 | 256061 | 153802 | 233900 | 106382 | 210287 | 95276 | 138272 |
| rno-let-7i-5p | 84824,19812 | 0,370242269 | 0,10094047 | 3,66792681 | 0,00024453 | 0,00141766 | UP | 90405,0583 | 59604,90164 | 77379,7721 | 74517,8541 | 63842,9784 | 92237,36 | 104486,115 | 87164,0469 | 103037,084 | 97070,2192 | 83320,7894 | 182562 | 88260 | 140097 | 66329 | 109967 | 55690 | 86828 |
| rno-miR-27a-3p | 168056,0681 | 0,36812542 | 0,10988347 | 3,35014381 | 0,0008077 | 0,00393348 | UP | 160467,716 | 125285,7754 | 158510,417 | 137600,028 | 143415,201 | 185107,413 | 179983,074 | 154827,963 | 215981,58 | 234532,4 | 152905,182 | 324045 | 185517 | 286985 | 122479 | 247027 | 111762 | 149566 |
| rno-miR-136-3p | 927,4299092 | 0,366388414 | 0,06505378 | 5,6320853 | 1,7804E-08 | 3,0967E-07 | UP | 850,261747 | 744,8924372 | 790,93652 | 767,3219 | 845,302469 | 1088,16566 | 1014,43999 | 990,368286 | 1019,29281 | 1070,53109 | 1020,2161 | 1717 | 1103 | 1432 | 683 | 1456 | 657 | 843 |
| rno-miR-505-3p | 915,8055703 | 0,361934564 | 0,06615504 | 5,47100533 | 4,4749E-08 | 6,8102E-07 | UP | 832,929679 | 740,8404385 | 781,546911 | 756,087319 | 844,141339 | 1076,57181 | 989,16924 | 957,782726 | 1019,29281 | 1061,1733 | 1014,3257 | 1682 | 1097 | 1415 | 673 | 1454 | 650 | 822 |
| rno-miR-200b-3p | 895,5302049 | 0,359933446 | 0,06533274 | 5,50923543 | 3,604E-08 | 5,8504E-07 | UP | 818,568823 | 727,3337759 | 765,529342 | 748,223112 | 812,21027 | 1060,00917 | 961,491755 | 934,507325 | 1013,58249 | 1016,25591 | 993,120288 | 1653 | 1077 | 1386 | 666 | 1399 | 640 | 799 |
| rno-miR-181a-5p | 110702,1804 | 0,358954104 | 0,10300429 | 3,48484601 | 0,00049242 | 0,00255116 | UP | 100431,907 | 82256,92533 | 118328,964 | 94133,433 | 84453,0317 | 136395,023 | 134719,556 | 99738,5819 | 120011,022 | 132321,011 | 114934,53 | 202810 | 121802 | 214236 | 83789 | 145467 | 82351 | 111952 |
| rno-miR-182 | 1371,112657 | 0,357194653 | 0,07430875 | 4,80689866 | 1,5329E-06 | 1,9142E-05 | UP | 1385,57505 | 1141,988315 | 1194,68973 | 1131,32233 | 1085,65633 | 1537,01329 | 1578,82 | 1402,34287 | 1537,5047 | 1575,85171 | 1511,47489 | 2798 | 1691 | 2163 | 1007 | 1870 | 928 | 1312 |
| rno-miR-133a-3p | 186258,9781 | 0,356039794 | 0,1102036 | 3,23074565 | 0,00123468 | 0,00585487 | UP | 172484,781 | 158513,5158 | 160832,965 | 167799,706 | 148305,299 | 227549,186 | 219013,141 | 159803,08 | 229856,239 | 245705,6 | 158985,246 | 348312 | 234719 | 291190 | 149360 | 255450 | 137387 | 182000 |
| rno-miR-455-5p | 1439,039286 | 0,354906191 | 0,0777802 | 4,56293766 | 5,0443E-06 | 5,5831E-05 | UP | 1481,64423 | 1148,066313 | 1275,88224 | 1198,72982 | 1136,16547 | 1616,51398 | 1625,75139 | 1482,643 | 1646,00086 | 1617,02598 | 1601,00886 | 2992 | 1700 | 2310 | 1067 | 1957 | 976 | 1351 |
| rno-miR-211-5p | 1205,385414 | 0,353716804 | 0,06051237 | 5,84536379 | 5,0546E-09 | 1,0257E-07 | UP | 1078,05464 | 1092,013664 | 1061,57821 | 990,890068 | 1004,37725 | 1348,19916 | 1303,24852 | 1253,38031 | 1351,91918 | 1414,89773 | 1360,68082 | 2177 | 1617 | 1922 | 882 | 1730 | 814 | 1083 |
| rno-miR-361-5p | 1405,713746 | 0,352190036 | 0,07856424 | 4,48282886 | 7,366E-06 | 7,6324E-05 | UP | 1454,90333 | 1144,014314 | 1257,10302 | 1157,16187 | 1089,13972 | 1560,20099 | 1587,24359 | 1477,98792 | 1566,05632 | 1615,15442 | 1553,88572 | 2938 | 1694 | 2276 | 1030 | 1876 | 942 | 1319 |
| rno-miR-203a-3p | 1342,483285 | 0,342830184 | 0,07103538 | 4,82618918 | 1,3917E-06 | 1,7836E-05 | UP | 1328,13163 | 1137,936316 | 1178,67216 | 1126,8285 | 1079,27012 | 1459,16887 | 1529,48188 | 1347,64568 | 1527,51164 | 1545,90678 | 1506,76257 | 2682 | 1685 | 2134 | 1003 | 1859 | 881 | 1271 |
| rno-miR-148a-3p | 1421,63655 | 0,340516061 | 0,0771514 | 4,41360824 | 1,0166E-05 | 9,7077E-05 | UP | 1472,7306 | 1146,715647 | 1269,25428 | 1183,00141 | 1130,35983 | 1561,85725 | 1611,31096 | 1479,15169 | 1590,3252 | 1617,02598 | 1576,26921 | 2974 | 1698 | 2298 | 1053 | 1947 | 943 | 1339 |
| rno-miR-122-3p | 880,2557691 | 0,336065554 | 0,06656503 | 5,04868009 | 4,4487E-07 | 6,0181E-06 | UP | 815,597611 | 726,6584428 | 764,977012 | 744,852738 | 791,8905 | 1048,41532 | 954,271542 | 891,447835 | 953,624087 | 1005,02656 | 986,051816 | 1647 | 1076 | 1385 | 663 | 1364 | 633 | 793 |
| rno-miR-29c-5p | 1229,977683 | 0,33144328 | 0,06322775 | 5,24205378 | 1,588E-07 | 2,2096E-06 | UP | 1140,45009 | 1102,14366 | 1122,3345 | 1009,98886 | 1008,4412 | 1381,32444 | 1361,01023 | 1266,18178 | 1356,20192 | 1418,64085 | 1363,03698 | 2303 | 1632 | 2032 | 899 | 1737 | 834 | 1131 |
| rno-miR-184 | 860,9546215 | 0,330426813 | 0,07323781 | 4,51169704 | 6,4311E-06 | 6,9599E-05 | UP | 811,140793 | 707,7491153 | 758,901382 | 736,988531 | 753,573217 | 1035,1652 | 927,797426 | 822,785403 | 947,913763 | 1001,28345 | 967,202558 | 1638 | 1048 | 1374 | 656 | 1298 | 625 | 771 |
| rno-miR-103-3p | 839,7629194 | 0,329953914 | 0,07446886 | 4,4307638 | 9,39E-06 | 9,1458E-05 | UP | 788,361504 | 658,4497972 | 751,721092 | 733,618157 | 744,864744 | 1008,66497 | 918,170475 | 816,966553 | 913,65182 | 976,953195 | 925,969806 | 1592 | 975 | 1361 | 653 | 1283 | 609 | 763 |
| rno-miR-652-3p | 33370,90925 | 0,32802537 | 0,09722469 | 3,37388952 | 0,00074114 | 0,00368302 | UP | 34137,7368 | 25979,39 | 31533,0704 | 25508,1168 | 29292,9817 | 35251,9298 | 34535,4842 | 37021,8518 | 40282,0522 | 42939,1516 | 30598,2363 | 68937 | 38469 | 57091 | 22705 | 50456 | 21284 | 28699 |
| rno-let-7d-5p | 119172,0155 | 0,326494067 | 0,10313731 | 3,16562521 | 0,0015475 | 0,00724647 | UP | 112720,344 | 94151,56766 | 121249,132 | 102130,208 | 93105,1902 | 146085,826 | 147896,445 | 101758,887 | 130192,529 | 143919,055 | 117682,987 | 227625 | 139415 | 219523 | 90907 | 160370 | 88202 | 122902 |
| rno-miR-486 | 125582,2143 | 0,326345994 | 0,09736085 | 3,35192226 | 0,00080253 | 0,00393348 | UP | 114631,328 | 96199,1777 | 121557,333 | 118084,437 | 101071,702 | 152154,378 | 151721,955 | 112225,834 | 148255,711 | 147800,666 | 117701,836 | 231484 | 142447 | 220081 | 105108 | 174092 | 91866 | 126081 |
| rno-miR-195-3p | 718,9014162 | 0,324789071 | 0,07950534 | 4,08512268 | 4,4054E-05 | 0,00033006 | UP | 692,787527 | 590,2411515 | 633,522478 | 587,5686 | 649,071538 | 864,569976 | 841,154865 | 678,477921 | 763,755818 | 810,384547 | 796,381156 | 1399 | 874 | 1147 | 523 | 1118 | 522 | 699 |
| rno-miR-143-3p | 105012,0562 | 0,324541299 | 0,10643084 | 3,0493164 | 0,00229363 | 0,01043922 | UP | 97646,8916 | 76022,24992 | 116328,977 | 87913,9688 | 83633,2741 | 123560,631 | 122064,928 | 95024,1496 | 118472,089 | 129571,693 | 104893,765 | 197186 | 112570 | 210615 | 78253 | 144055 | 74602 | 101436 |
| rno-miR-92b-3p | 751,9740283 | 0,324074371 | 0,07661084 | 4,23013744 | 2,3355E-05 | 0,00019954 | UP | 715,071615 | 607,7998128 | 695,935765 | 610,037763 | 669,971874 | 887,757676 | 874,849194 | 732,011342 | 803,728085 | 853,430377 | 821,120808 | 1444 | 900 | 1260 | 543 | 1154 | 536 | 727 |
| rno-miR-6331 | 814,0905183 | 0,323227098 | 0,0718332 | 4,49968975 | 6,8053E-06 | 7,2047E-05 | UP | 774,495849 | 648,9951334 | 716,924304 | 715,642827 | 719,900454 | 977,19595 | 912,15363 | 806,492623 | 900,803591 | 896,476208 | 885,915132 | 1564 | 961 | 1298 | 637 | 1240 | 590 | 758 |
| rno-miR-19b-3p | 1478,392477 | 0,322722235 | 0,07514888 | 4,29443845 | 1,7514E-05 | 0,00015508 | UP | 1495,50989 | 1293,938268 | 1348,23746 | 1211,08786 | 1148,35734 | 1699,32719 | 1640,19182 | 1518,71987 | 1677,40764 | 1622,64065 | 1606,89926 | 3020 | 1916 | 2441 | 1078 | 1978 | 1026 | 1363 |
| rno-miR-22-5p | 823,425806 | 0,320273755 | 0,07170155 | 4,46676189 | 7,9412E-06 | 8,0571E-05 | UP | 781,923878 | 653,7224653 | 721,342944 | 732,494699 | 733,253446 | 977,19595 | 918,170475 | 813,475243 | 903,658753 | 896,476208 | 925,969806 | 1579 | 968 | 1306 | 652 | 1263 | 590 | 763 |
| rno-miR-484 | 1307,461551 | 0,316175192 | 0,07546667 | 4,18960048 | 2,7945E-05 | 0,00022682 | UP | 1324,66521 | 1117,676322 | 1165,96857 | 1072,90251 | 1079,27012 | 1409,48094 | 1492,17744 | 1277,81948 | 1439,00162 | 1523,44809 | 1479,66676 | 2675 | 1655 | 2111 | 955 | 1859 | 851 | 1240 |
| rno-miR-9a-5p | 738,8173478 | 0,315657699 | 0,07723444 | 4,08700693 | 4,3697E-05 | 0,00033006 | UP | 697,739547 | 605,0984803 | 678,813536 | 608,914305 | 662,424531 | 879,476355 | 858,002029 | 698,262011 | 790,879856 | 830,971683 | 816,408493 | 1409 | 896 | 1229 | 542 | 1141 | 531 | 713 |
| rno-miR-7a-1-3p | 1280,767336 | 0,315403041 | 0,0688954 | 4,57799827 | 4,6945E-06 | 5,4433E-05 | UP | 1250,88012 | 1106,870992 | 1160,9976 | 1050,43335 | 1074,6256 | 1401,19962 | 1475,33028 | 1273,1644 | 1430,43613 | 1467,30135 | 1397,20126 | 2526 | 1639 | 2102 | 935 | 1851 | 846 | 1226 |
| rno-miR-326-3p | 764,3982378 | 0,313198568 | 0,07625113 | 4,10746111 | 4,0003E-05 | 0,00031422 | UP | 753,697367 | 613,2024778 | 695,935765 | 634,753842 | 672,294134 | 887,757676 | 883,272776 | 757,614282 | 805,155666 | 855,301935 | 849,394695 | 1522 | 908 | 1260 | 565 | 1158 | 536 | 734 |
| rno-miR-200c-3p | 801,5772821 | 0,310502687 | 0,07046783 | 4,40630419 | 1,0515E-05 | 9,8476E-05 | UP | 773,010243 | 648,9951334 | 714,162654 | 694,297122 | 708,869721 | 942,414399 | 904,933417 | 801,837543 | 887,955362 | 870,274398 | 870,60011 | 1561 | 961 | 1293 | 618 | 1221 | 569 | 752 |
| rno-let-7e-3p | 785,4190726 | 0,309774507 | 0,07301153 | 4,24281607 | 2,2073E-05 | 0,00019196 | UP | 768,553426 | 631,4364722 | 706,430034 | 675,198334 | 687,388821 | 925,851756 | 897,713203 | 789,036073 | 837,990028 | 855,301935 | 864,709717 | 1552 | 935 | 1279 | 601 | 1184 | 559 | 746 |
| rno-miR-376b-5p | 728,0554778 | 0,308982361 | 0,07696061 | 4,01481167 | 5,9493E-05 | 0,0004199 | UP | 694,768335 | 592,942484 | 665,005287 | 607,790846 | 654,296622 | 866,22624 | 843,561602 | 692,443161 | 775,176466 | 814,127662 | 802,271549 | 1403 | 878 | 1204 | 541 | 1127 | 523 | 701 |
| rno-miR-146a-5p | 131869,6675 | 0,307538789 | 0,09521595 | 3,22990816 | 0,0012383 | 0,00585487 | UP | 116012,446 | 103411,7355 | 124414,536 | 118191,165 | 121667,241 | 152492,256 | 157324,841 | 116264,116 | 155627,739 | 163882,963 | 121277,305 | 234273 | 153127 | 225254 | 105203 | 209567 | 92070 | 130737 |
| rno-miR-219a-2-3p | 608,3394487 | 0,304099149 | 0,07517294 | 4,04532733 | 5,225E-05 | 0,00037979 | UP | 582,357493 | 534,8638352 | 514,771534 | 521,284571 | 538,183646 | 660,849464 | 703,970809 | 584,21255 | 656,687245 | 733,650675 | 660,902113 | 1176 | 792 | 932 | 464 | 927 | 399 | 585 |
| rno-miR-125b-2-3p | 794,6851692 | 0,303915888 | 0,07201357 | 4,22025866 | 2,4402E-05 | 0,00020489 | UP | 772,019839 | 640,8911359 | 711,401004 | 688,679832 | 705,386332 | 937,445606 | 902,526679 | 791,363613 | 859,403743 | 866,531282 | 865,887796 | 1559 | 949 | 1288 | 613 | 1215 | 566 | 750 |
| rno-miR-125b-1-3p | 1252,377839 | 0,30371021 | 0,06848386 | 4,43477043 | 9,217E-06 | 9,1458E-05 | UP | 1235,03366 | 1102,14366 | 1130,61945 | 1018,97652 | 1056,62809 | 1397,88709 | 1361,01023 | 1267,34555 | 1373,33289 | 1465,42979 | 1367,74929 | 2494 | 1632 | 2047 | 907 | 1820 | 844 | 1131 |
| rno-miR-187-3p | 1521,14128 | 0,303527325 | 0,07455182 | 4,07136038 | 4,6739E-05 | 0,00034488 | UP | 1514,32756 | 1334,458256 | 1420,59269 | 1261,64348 | 1207,57496 | 1806,98437 | 1651,02214 | 1521,04741 | 1710,242 | 1628,25532 | 1676,4059 | 3058 | 1976 | 2572 | 1123 | 2080 | 1091 | 1372 |
| rno-miR-27b-3p | 90445,48745 | 0,302109231 | 0,10725856 | 2,81664452 | 0,00485282 | 0,02091437 | UP | 91697,0402 | 64237,01155 | 98998,5196 | 74853,7681 | 71453,0229 | 102141,821 | 104912,108 | 87684,2521 | 106234,865 | 106910,87 | 85777,0834 | 185171 | 95119 | 179238 | 66628 | 123075 | 61670 | 87182 |
| rno-miR-92a-3p | 99260,98926 | 0,298160173 | 0,10137415 | 2,94118533 | 0,00326959 | 0,01447536 | UP | 97066,5149 | 74854,59894 | 107365,214 | 82907,8394 | 78872,6421 | 114267,332 | 117037,253 | 92003,0026 | 114686,145 | 117354,163 | 95456,1774 | 196014 | 110841 | 194386 | 73797 | 135855 | 68991 | 97258 |
| rno-let-7a-5p | 94232,54096 | 0,295760698 | 0,1055419 | 2,80230605 | 0,00507387 | 0,02167522 | UP | 92480,9449 | 72861,01556 | 105227,697 | 75143,6203 | 73423,4601 | 111883,967 | 105807,414 | 88637,3797 | 109748,142 | 111552,334 | 89791,9754 | 186754 | 107889 | 190516 | 66886 | 126469 | 67552 | 87926 |
| rno-miR-322-3p | 674,9561824 | 0,294073715 | 0,07952679 | 3,69779452 | 0,00021748 | 0,00128277 | UP | 667,532228 | 585,5138196 | 578,289481 | 566,222896 | 600,884653 | 766,850381 | 791,81674 | 624,9445 | 730,921456 | 782,311179 | 729,230674 | 1348 | 867 | 1047 | 504 | 1035 | 463 | 658 |
| rno-miR-411-3p | 621,9663844 | 0,293374534 | 0,07447152 | 3,93941914 | 8,1679E-05 | 0,0005449 | UP | 604,146378 | 544,9938321 | 524,713474 | 542,630275 | 548,053249 | 680,724636 | 711,191023 | 598,17779 | 678,10096 | 737,393791 | 671,504821 | 1220 | 807 | 950 | 483 | 944 | 411 | 591 |
| rno-miR-15a-5p | 597,0806872 | 0,292063766 | 0,07145848 | 4,08718157 | 4,3665E-05 | 0,00033006 | UP | 567,006232 | 526,7598377 | 511,457554 | 516,790738 | 532,958562 | 645,943085 | 670,27648 | 581,885009 | 648,12176 | 709,320423 | 657,367878 | 1145 | 780 | 926 | 460 | 918 | 390 | 557 |
| rno-miR-181a-1-3p | 661,537648 | 0,289907162 | 0,08043699 | 3,60415208 | 0,00031317 | 0,00177344 | UP | 659,608997 | 566,6044921 | 564,481232 | 554,988315 | 597,401264 | 760,225323 | 755,715672 | 612,14303 | 726,638713 | 771,081832 | 708,025259 | 1332 | 839 | 1022 | 494 | 1029 | 459 | 628 |
| rno-miR-410-3p | 698,5602833 | 0,288650436 | 0,08085446 | 3,57000027 | 0,00035698 | 0,00197556 | UP | 687,835508 | 588,8904853 | 607,562969 | 583,074768 | 643,846455 | 778,444231 | 825,511069 | 641,23728 | 742,342104 | 797,283642 | 788,134606 | 1389 | 872 | 1100 | 519 | 1109 | 470 | 686 |
| rno-miR-323-3p | 583,4447104 | 0,287016799 | 0,06810974 | 4,21403438 | 2,5085E-05 | 0,00020706 | UP | 538,779721 | 503,7985115 | 509,800564 | 516,790738 | 531,797432 | 627,724178 | 641,395626 | 579,557469 | 636,701112 | 681,247055 | 650,299406 | 1088 | 746 | 923 | 460 | 916 | 379 | 533 |
| rno-miR-200a-3p | 1581,895889 | 0,284537868 | 0,07697571 | 3,69646305 | 0,00021862 | 0,00128277 | UP | 1589,10306 | 1348,640251 | 1483,55831 | 1402,07574 | 1242,40885 | 1906,36023 | 1724,42764 | 1593,20116 | 1793,0417 | 1635,74156 | 1682,29629 | 3209 | 1997 | 2686 | 1248 | 2140 | 1151 | 1433 |
| rno-miR-212-3p | 647,49488 | 0,283194948 | 0,08068742 | 3,5097784 | 0,00044848 | 0,00240525 | UP | 656,637785 | 563,9031596 | 561,719582 | 553,864856 | 553,858898 | 703,912337 | 754,512303 | 603,99664 | 723,783551 | 752,366253 | 693,888315 | 1326 | 835 | 1017 | 493 | 954 | 425 | 627 |
| rno-miR-493-3p | 689,2015001 | 0,282647808 | 0,07892969 | 3,58100742 | 0,00034227 | 0,00191594 | UP | 673,969853 | 587,539819 | 599,83035 | 574,087103 | 640,94363 | 778,444231 | 797,833584 | 635,41843 | 736,63178 | 786,054295 | 770,463426 | 1361 | 870 | 1086 | 511 | 1104 | 470 | 663 |
| rno-miR-138-5p | 1549,860914 | 0,280919124 | 0,07442251 | 3,77465274 | 0,00016023 | 0,00101341 | UP | 1547,01089 | 1341,88692 | 1448,76152 | 1381,8535 | 1213,3806 | 1858,32857 | 1659,44572 | 1571,08953 | 1715,95232 | 1631,99844 | 1678,76205 | 3124 | 1987 | 2623 | 1230 | 2090 | 1122 | 1379 |
| rno-miR-142-5p | 637,6607897 | 0,276261886 | 0,07891692 | 3,50066742 | 0,00046409 | 0,00245667 | UP | 641,286525 | 556,4744952 | 557,853272 | 546,00065 | 552,117203 | 695,631015 | 746,088721 | 602,83287 | 688,094027 | 750,494696 | 677,395214 | 1295 | 824 | 1010 | 486 | 951 | 420 | 620 |
| rno-miR-3068-5p | 574,2727757 | 0,274356115 | 0,06856138 | 4,00161314 | 6,2912E-05 | 0,00043769 | UP | 534,322904 | 503,7985115 | 495,992315 | 511,173448 | 528,314043 | 607,849006 | 634,175413 | 573,738619 | 622,425302 | 673,760824 | 631,450148 | 1079 | 746 | 898 | 455 | 910 | 367 | 527 |
| rno-miR-503-3p | 1603,215167 | 0,267040406 | 0,07212686 | 3,70237109 | 0,00021359 | 0,00128277 | UP | 1591,57907 | 1354,718249 | 1490,7386 | 1451,5079 | 1324,2685 | 1929,54793 | 1743,68154 | 1606,00263 | 1803,03476 | 1654,45713 | 1685,83052 | 3214 | 2006 | 2699 | 1292 | 2281 | 1165 | 1449 |
| rno-miR-146b-5p | 1800,035197 | 0,262846437 | 0,06384385 | 4,11702048 | 3,838E-05 | 0,00030641 | UP | 1758,95732 | 1553,266188 | 1658,64691 | 1605,42166 | 1535,59411 | 2133,26845 | 1936,22057 | 1994,70181 | 1878,69655 | 1908,989 | 1836,62459 | 3552 | 2300 | 3003 | 1429 | 2645 | 1288 | 1609 |
| rno-miR-139-5p | 27526,11747 | 0,259648678 | 0,08859557 | 2,93071847 | 0,00338179 | 0,01483723 | UP | 27738,7372 | 23305,07082 | 27368,5025 | 24486,8933 | 21363,046 | 28065,3989 | 33399,504 | 28949,943 | 31505,2844 | 31518,9057 | 25086,0064 | 56015 | 34509 | 49551 | 21796 | 36797 | 16945 | 27755 |
| rno-miR-493-5p | 1676,574101 | 0,252417412 | 0,06796276 | 3,71405454 | 0,00020396 | 0,00125735 | UP | 1619,31037 | 1420,900896 | 1599,5476 | 1472,8536 | 1471,73198 | 2047,1427 | 1768,95229 | 1708,41439 | 1813,02783 | 1800,43865 | 1719,99481 | 3270 | 2104 | 2896 | 1311 | 2535 | 1236 | 1470 |
| rno-let-7f-1-3p | 1974,445894 | 0,251398248 | 0,06531415 | 3,84906271 | 0,00011857 | 0,00075979 | UP | 1875,82498 | 1749,112795 | 1851,41007 | 1713,27364 | 1746,91973 | 2336,98896 | 2198,55499 | 2051,72654 | 2158,50242 | 2141,06217 | 1895,52852 | 3788 | 2590 | 3352 | 1525 | 3009 | 1411 | 1827 |
| rno-miR-150-5p | 30855,53633 | 0,247356737 | 0,07423906 | 3,33189499 | 0,00086257 | 0,00415911 | UP | 31588,4372 | 24985,97497 | 29258,0233 | 25430,5982 | 28724,6087 | 32459,4682 | 34376,6395 | 32756,6347 | 34767,3069 | 35091,7096 | 29971,4985 | 63789 | 36998 | 52972 | 22636 | 49477 | 19598 | 28567 |
| rno-miR-425-3p | 2032,971916 | 0,247122246 | 0,07212632 | 3,42624207 | 0,00061199 | 0,0031046 | UP | 1968,42775 | 1753,840126 | 1906,64306 | 1811,0145 | 1777,68967 | 2491,02154 | 2203,36847 | 2054,05408 | 2205,6126 | 2258,97032 | 1932,04896 | 3975 | 2597 | 3452 | 1612 | 3062 | 1504 | 1831 |
| rno-miR-350 | 1726,899737 | 0,246500059 | 0,06582251 | 3,74492044 | 0,00018045 | 0,00112666 | UP | 1628,71921 | 1528,954196 | 1642,62934 | 1533,52034 | 1497,8574 | 2110,08075 | 1855,59485 | 1724,70717 | 1847,28977 | 1854,71382 | 1771,83026 | 3289 | 2264 | 2974 | 1365 | 2580 | 1274 | 1542 |
| rno-miR-222-3p | 1929,143158 | 0,245223401 | 0,0610235 | 4,01850761 | 5,8568E-05 | 0,00041945 | UP | 1866,91135 | 1690,358813 | 1756,96164 | 1711,02673 | 1727,76109 | 2221,05045 | 2032,49008 | 2040,08884 | 2152,7921 | 2127,96127 | 1893,17236 | 3770 | 2503 | 3181 | 1523 | 2976 | 1341 | 1689 |
| rno-miR-221-3p | 1644,213477 | 0,238924766 | 0,06991461 | 3,41737951 | 0,00063227 | 0,00317439 | UP | 1611,38714 | 1419,550229 | 1588,501 | 1461,61902 | 1399,16137 | 1980,89213 | 1744,88491 | 1625,78672 | 1810,17267 | 1725,57633 | 1718,81673 | 3254 | 2102 | 2876 | 1301 | 2410 | 1196 | 1450 |
| rno-miR-466b-5p | 559,826237 | 0,238217879 | 0,06788363 | 3,5092093 | 0,00044944 | 0,00240525 | UP | 533,3325 | 493,6685146 | 495,992315 | 511,173448 | 511,477661 | 601,223949 | 588,447395 | 562,100919 | 619,57014 | 643,815899 | 597,285867 | 1077 | 731 | 898 | 455 | 881 | 363 | 489 |
| rno-miR-199a-5p | 1863,708583 | 0,234057774 | 0,05959509 | 3,92746761 | 8,5845E-05 | 0,00056495 | UP | 1788,17424 | 1641,734828 | 1729,34514 | 1621,15008 | 1713,82753 | 2177,98758 | 1996,38901 | 1998,19312 | 1970,06174 | 2002,56689 | 1861,36424 | 3611 | 2431 | 3131 | 1443 | 2952 | 1315 | 1659 |
| rno-miR-126a-5p | 1749,866286 | 0,231305589 | 0,06663789 | 3,47108188 | 0,00051837 | 0,00265731 | UP | 1691,11466 | 1537,733526 | 1648,70497 | 1596,434 | 1512,37152 | 2128,29965 | 1864,01843 | 1724,70717 | 1847,28977 | 1865,94317 | 1831,91228 | 3415 | 2277 | 2985 | 1421 | 2605 | 1285 | 1549 |
| rno-miR-489-5p | 534,8443591 | 0,217833593 | 0,07017209 | 3,10427673 | 0,00190745 | 0,00884693 | UP | 516,000431 | 488,2658496 | 469,480476 | 480,840078 | 496,382974 | 581,348777 | 549,93959 | 545,808139 | 546,763511 | 628,843436 | 579,614688 | 1042 | 723 | 850 | 428 | 855 | 351 | 457 |
| rno-miR-196b-5p | 1891,990325 | 0,217692601 | 0,05877042 | 3,70411846 | 0,00021213 | 0,00128277 | UP | 1822,34317 | 1680,904149 | 1751,99067 | 1703,16252 | 1724,2777 | 2211,11287 | 2021,65976 | 2029,61491 | 1981,48238 | 2006,31001 | 1879,03542 | 3680 | 2489 | 3172 | 1516 | 2970 | 1335 | 1680 |
| rno-miR-181d-5p | 2079,074034 | 0,214672233 | 0,07792734 | 2,75477416 | 0,00587327 | 0,02465759 | UP | 2046,17446 | 1793,684781 | 2083,38866 | 1849,21208 | 1779,43136 | 2542,36573 | 2225,02911 | 2071,51063 | 2252,72277 | 2268,32811 | 1957,96669 | 4132 | 2656 | 3772 | 1646 | 3065 | 1535 | 1849 |
| rno-miR-133b-3p | 29176,02176 | 0,213818509 | 0,0785616 | 2,72166702 | 0,00649536 | 0,02703622 | UP | 30594,5668 | 24131,00323 | 28804,5604 | 25207,03 | 25444,4171 | 29933,6651 | 34178,0836 | 30161,4276 | 31672,3114 | 33012,4088 | 27796,7654 | 61782 | 35732 | 52151 | 22437 | 43827 | 18073 | 28402 |
| rno-miR-301a-3p | 544,9910078 | 0,210752317 | 0,06909484 | 3,05018903 | 0,00228697 | 0,01043922 | UP | 528,38048 | 493,6685146 | 486,050376 | 487,580827 | 508,574837 | 581,348777 | 560,76991 | 551,626989 | 573,887549 | 636,329667 | 586,68316 | 1067 | 731 | 880 | 434 | 876 | 351 | 466 |
| rno-miR-183-5p | 2151,927668 | 0,206915607 | 0,0744248 | 2,7801972 | 0,00543259 | 0,02300584 | UP | 2050,13607 | 1860,54276 | 2098,30156 | 2026,71846 | 1883,35248 | 2592,05366 | 2226,23247 | 2235,60221 | 2305,54326 | 2421,79585 | 1970,92555 | 4140 | 2755 | 3799 | 1804 | 3244 | 1565 | 1850 |
| rno-miR-193b-3p | 2437,215002 | 0,199976365 | 0,06795034 | 2,94297827 | 0,00325071 | 0,01447536 | UP | 2336,3628 | 2192,131325 | 2354,58267 | 2322,18795 | 2062,74703 | 2953,11928 | 2511,4309 | 2474,17506 | 2553,94235 | 2618,30942 | 2430,37622 | 4718 | 3246 | 4263 | 2067 | 3553 | 1783 | 2087 |
| rno-miR-24-2-5p | 521,8271586 | 0,198698291 | 0,06917525 | 2,87238996 | 0,0040738 | 0,01771375 | UP | 490,745132 | 486,2398502 | 467,271157 | 472,975871 | 494,060715 | 549,879755 | 541,516007 | 537,661749 | 519,639472 | 606,384742 | 573,724295 | 991 | 720 | 846 | 421 | 851 | 332 | 450 |
| rno-miR-1193-3p | 513,5674886 | 0,181704724 | 0,06874027 | 2,64335192 | 0,00820897 | 0,03359468 | UP | 488,764324 | 483,5385177 | 461,747857 | 467,358581 | 488,835631 | 539,942169 | 522,262105 | 537,661749 | 512,501568 | 576,439816 | 570,190059 | 987 | 716 | 836 | 416 | 842 | 326 | 434 |
| rno-let-7e-5p | 2496,059555 | 0,17500814 | 0,06781292 | 2,58074903 | 0,00985862 | 0,04000958 | UP | 2383,90218 | 2195,50799 | 2465,04867 | 2416,55843 | 2195,11582 | 3001,15095 | 2548,73534 | 2532,36356 | 2605,33527 | 2661,35525 | 2451,58164 | 4814 | 3251 | 4463 | 2151 | 3781 | 1812 | 2118 |
| rno-miR-205 | 2553,042399 | 0,169101426 | 0,06606658 | 2,55956095 | 0,01048045 | 0,04218164 | UP | 2394,30143 | 2245,482642 | 2577,17165 | 2482,84246 | 2251,43061 | 3017,71359 | 2620,93748 | 2557,9665 | 2651,01786 | 2708,1442 | 2576,45797 | 4835 | 3325 | 4666 | 2210 | 3878 | 1822 | 2178 |
| rno-miR-3544 | 35,17700095 | -0,465032489 | 0,18719795 | -2,48417511 | 0,01298519 | 0,0494046 | DOWN | 41,5969638 | 43,89665314 | 39,767758 | 40,4444926 | 41,8006715 | 24,8439648 | 30,0842226 | 32,5855605 | 27,1240384 | 31,8164834 | 32,9862017 | 84 | 65 | 72 | 36 | 72 | 15 | 25 |
| rno-miR-29a-5p | 33,50894729 | -0,478900988 | 0,19173944 | -2,49766546 | 0,01250141 | 0,04793848 | DOWN | 41,1017618 | 42,54598689 | 37,0061081 | 38,1975763 | 39,478412 | 24,8439648 | 28,8808537 | 29,0942505 | 25,6964574 | 29,9449255 | 31,8081231 | 83 | 63 | 67 | 34 | 68 | 15 | 24 |
| rno-miR-802-5p | 29,32122131 | -0,514552125 | 0,20424822 | -2,51924897 | 0,01176055 | 0,04694579 | DOWN | 36,1497423 | 37,14332189 | 34,2444583 | 31,4568275 | 35,9950227 | 23,1877005 | 22,8640092 | 25,6029404 | 21,4137145 | 26,2018098 | 28,2738872 | 73 | 55 | 62 | 28 | 62 | 14 | 19 |

**Table S3**

**Tab. 3 . Results of miRs target research in the TAA rat group**

| **miRNA** | **Independent databases** | | |
| --- | --- | --- | --- |
|  | **miRDB** | **TargetScan** | **TaRBase** |
| rno-let-7c-5p | integrin subunit alpha L, claudin 12 | integrin, beta 3, integrin, beta 8, claudin 12, protocadherin 19, cell adhesion molecule 2, cadherin 22, collagen, type IV alpha 2, collagen type 24 alpha 1, collagen type 15 alpha 1, collagen, type 9 alpha 3, collagen type 4 alpha 5, collagen, type 5 alpha 2, collagen, type 4 alpha 3, fibronectin type 3, collagen, type 3 alpha 1, claudin 23 | laminin c1 |
| rno-miR-16-5p | claudin 12 | integrin, alpha 2, claudin 12, protocadherin alpha 13, protocadherin alpha 4, protocadherin alpha 11, protocadherin alpha 9, protocadherin alpha 6 protocadherin alpha 1, protocadherin alpha 8, protocadherin alpha 2, collagen type 24 alpha 1, laminin gamma 1, claudin 2, protocadherin 1, cell adhesion molecule 1 | claudin 11 |
| rno-miR-369-5p | - | Integrin alpha 1, claudin 1 | - |
| rno-miR-206-3p | gap junction protein beta 2, protocadherin 17 | gap junction protein, alpha 1 | - |
| rno-miR-149-5p | gap junction protein, gamma 2, integrin subunit alpha 5, laminin subunit gamma 3 | protocadherin 17 | - |
| rno-let-7f-5p | integrin subunit alpha L, collagen type V alpha 2 chain, collagen type V alpha 2 chain | claudin 12, integrin, beta 3, cadherin 22 type 2, integrin beta 8, claudin 23, gap junction protein alpha 9, cell adhesion molecule 2 | - |
| rno-miR-30d-5p | protocadherin 17, collagen type XIII alpha 1 chain, gap junction protein, alpha 1, integrin subunit alpha 6, integrin subunit alpha 8, protocadherin 20, claudin domain containing 1 | cell adhesion molecule 2, cell adhesion molecule L1-like, cell adhesion molecule 2, cell adhesion molecule 1, integrin, alpha 9, integrin alpha 8, integrin alpha 6, gap junction protein, gap junction protein alpha 1, claudin 19, protocadherin 17, protocadherin 20, protocadherin 19 | protocadherin 17, gap junction protein alpha 2, protocadherin 20 |
| rno-miR-126a-3p |  | integrin, alpha 6, protocadherin 7 | - |
| rno-miR-429 | fibronectin 1, cadherin 11, protocadherin 18, laminin subunit gamma 1 | protocadherin 8, protocadherin 19, cell adhesion molecule 1, gap junction protein, gamma 1 | protocadherin 19 |
| rno-miR-3559-3p | collagen type XVII alpha 1 chain, integrin subunit alpha 8, laminin subunit gamma 3, | integrin, beta 8, integrin, alpha 8, integrin, alpha 1, cell adhesion molecule 1, gap junction protein, gamma 2, protocadherin 7, protocadherin 10, protocadherin 18 | - |
| rno-miR-214-3p | - | protocadherin 20, protocadherin 19, protocadherin beta 16, protocadherin beta 2, integrin alpha 1, integrin, beta 5, integrin alpha 8, integrin beta 8, integrin alpha 9 | - |
| rno-miR-10a-5p | occludin | integrin, beta 8, cell adhesion molecule 2, cell adhesion molecule L1-like, gap junction protein alpha 9 | - |
| rno-miR-100-5p | - | - | - |
| rno-miR-455-3p | endothelial cell-specific molecule 1, catenin delta 1, claudin 9, | integrin beta 1 | - |
| rno-miR-449a-5p | endothelin 2, cadherin 9, gap junction protein, beta 2, | cell adhesion molecule L1-like | - |
| rno-miR-23a-3p | protocadherin 17, claudin 12, gap junction protein, alpha 1, catenin, beta-interacting protein 1, collagen type IV alpha 1 chain, protocadherin 18, tight junction protein 2 | integrin beta 8, integrin alpha 1, cell adhesion molecule 2, cell adhesion molecule 1, protocadherin 19, gap junction protein alpha 1 | claudin 12 |
| rno-miR-122-5p | occludin | occludin, cell adhesion molecule 2, protocadherin 7 | occludin |
| rno-miR-99b-3p | claudin 2 | protocadherin 1, protocadherin 9 | - |
| rno-miR-337-5p | - | junctional adhesion molecule 3, integrin beta 1 binding protein 1 | - |
| rno-let-7g-5p | collagen type IV alpha 1 chain, endothelin 1, claudin 12, integrin subunit alpha L, collagen type V alpha 2 chain | integrin, beta 3, integrin, beta 8, cell adhesion molecule 2, protocadherin 19 | - |
| rno-miR-99a-5p | - | - | - |
| rno-miR-130a-3p | gap junction protein, alpha 1, endothelial cell-specific molecule 1 | gap junction protein, alpha 1, 43kDa, integrin, beta 8 | collagen type 16 alpha chain 1 |
| rno-miR-380-3p | - | integrin, alpha 1, cell adhesion molecule 3 | - |
| rno-miR-211-5p | - | integrin, alpha 11 | - |
| rno-miR-466c-5p | - | claudin 23, claudin 18 | - |
| rno-miR-329-3p | - | protocadherin beta 16, protocadherin alpha 5, gap junction protein alpha 1, protocadherin 17 | - |
| rno-miR-1-3p | gap junction protein, alpha 1, gap junction protein beta 2, protocadherin 17, integrin subunit alpha 11 | - | - |
| rno-miR-136-3p | - | claudin domain containing 1, integrin alpha 11, protocadherin alpha 11 | - |
| rno-miR-379-3p | cadherin 2, integrin subunit beta 1, protocadherin 20 | cell adhesion molecule 1 | - |
| rno-miR-200b-3p | cadherin 11, claudin 12, protocadherin 19 | occludin | - |
| rno-miR-341 | - | - | - |
| rno-miR-505-3p | protocadherin 9, cadherin 9, cadherin 8, collagen type 11 alpha 1 chain | - | - |
| rno-let-7b-5p | collagen type IV alpha 1 chain, claudin 12, integrin subunit alpha L | integrin, beta 8, claudin 12, claudin 23, collagen, type 27 alpha 1 | - |
| rno-miR-497-5p | laminin subunit gamma 1, protocadherin 9, claudin 12 | claudin 12, claudin 2, integrin alpha 2 | claudin 12 |
| rno-miR-29c-5p | protocadherin 19 | claudin 12, protocadherin 19 | - |
| rno-miR-122-3p | - | integrin alpha 8 | - |
| rno-miR-22-3p | cadherin 16 | cell adhesion molecule 1 | - |
| rno-miR-203a-3p | cadherin 10, collagen type XIV alpha 1 chain, gap junction protein, alpha 3 | integrin alpha 2 | - |
| rno-miR-182 | protocadherin 18 | protocadherin 8 | laminin c1 |
| rno-miR-93-5p | cerebral endothelial cell adhesion molecule | integrin beta 8 | - |
| rno-miR-26b-5p | protocadherin alpha 4 | integrin beta 8, occludin, gap junction protein alpha 9 | - |
| rno-miR-7a-1-3p | protocadherin 9, collagen type XVI alpha 1 chain, collagen type V alpha 2 chain, protocadherin 9, laminin subunit gamma 2, claudin 12 | occludin, integrin beta 8, gap junction protein, alpha 9 | - |
| rno-miR-192-5p | - | - | collagen, type 1 alpha 1 |
| rno-miR-455-5p | tight junction protein 2, protocadherin alpha 4 | tight junction protein 1 | - |
| rno-miR-184 | - | - | - |
| rno-miR-6331 | protocadherin beta 6, junctional adhesion molecule 2 | gap junction protein alpha 3, integrin, beta 4, cadherin 2, type 1, claudin 5, gap junction protein, alpha 3, adherens junctions associated protein 1 | - |
| rno-miR-361-5p | collagen type XI alpha 1 chain, tight junction protein 2 | claudin 22, cadherin 20, type 2, gap junction protein alpha 9 | - |
| rno-miR-22-5p | syndecan 1 | adherens junctions associated protein 1, integrin beta 1, claudin 2, junctional adhesion molecule 2, gap junction protein gamma 1 | - |
| rno-miR-125b-1-3p | - | integrin alpha 6, cadherin 10 type 2 | - |
| rno-miR-103-3p | - | - | - |
| rno-miR-148a-3p | collagen type 4 alpha 1 chain, integrin subunit alpha 11, gap junction protein, delta 1 | integrin alpha 9, gap junction protein delta 2 | - |
| rno-miR-200c-3p | fibronectin 1, cadherin 11, claudin 12, protocadherin 19 | occludin, cadherin 20 type 2, gap junction protein gamma 1 | - |
| rno-miR-191a-5p | - | adherens junctions associated protein 1 | - |
| rno-miR-451-5p | - | - | - |
| rno-miR-19b-3p | protocadherin alpha 4 | protocadherin alpha 5, cell adhesion molecule L1-like | - |
| rno-let-7e-3p | - | integrin, beta 2 | - |
| rno-miR-92b-3p | protocadherin 9, claudin 2 | integrin alpha 5 | - |
| rno-miR-125b-2-3p | claudin 16, occludin, claudin 19 | integrin beta 1 , claudin 11, junctional adhesion molecule 2 | - |
| rno-miR-323-3p | claudin 1 | - | - |
| rno-miR-484 | dystroglycan 1, laminin subunit beta 3, protocadherin gamma subfamily A, 1 | integrin beta 2, claudin 12 | - |
| rno-miR-146b-5p | - | - | - |
| rno-miR-326-3p | dystroglycan 1, protocadherin 9 | gap junction protein alpha 9, cadherin 22 type 2 | - |
| rno-miR-9a-5p | collagen type 15 alpha 1 chain | protocadherin 7, integrin alpha 6 | integrin, beta 1 |
| rno-miR-195-3p | - | claudin 16, cadherin 7 type 2 | - |
| rno-miR-15a-5p | protocadherin alpha 4 | claudin 12, integrin alpha 2 | - |
| rno-miR-187-3p | gap junction protein, alpha 1 | - | - |
| rno-miR-219a-2-3p | - | - | - |
| rno-miR-222-3p | adherens junctions associated protein 1 | Integrin beta 8, protocadherin alpha 3, adherens junctions associated protein 1 | adherens junctions associated protein 1 |
| rno-miR-376b-5p | - | integrin alpha 10, gap junction protein beta 3 | - |
| rno-miR-3068-5p | - | integrin beta 6, occludin | - |
| rno-miR-26a-5p | claudin domain containing 1, cadherin 11, junctional adhesion molecule 2, cadherin 2 | occludin, integrin beta 8, gap junction protein alpha 9 | - |
| rno-miR-127-3p | - | - | - |
| rno-miR-411-3p | - | tight junction protein 1, cadherin 2 type 1, N-cadherin | - |
| rno-miR-199a-5p | collagen type V alpha 3 chain | cadherin 8, type 2 | - |
| rno-miR-24-3p | collagen type XI alpha 2 chain | protocadherin 10 | - |
| rno-let-7f-1-3p | claudin 12, claudin 1, integrin subunit alpha 6, tight junction protein 1 | integrin alpha 5, adherens junctions associated protein 1 | - |
| rno-miR-138-5p | integrin subunit alpha L | - | - |
| rno-miR-350 | - | integrin beta 8, occludin, claudin 1 | - |
| rno-miR-493-5p | integrin subunit beta 1 | cadherin 2 type 1, N-cadherin | - |
| rno-miR-196b-5p | - | cell adhesion molecule 2 | - |
| rno-miR-503-3p | - | cadherin 2 type 1, N-cadherin, gap junction protein, gamma 1 | - |
| rno-miR-200a-3p | protocadherin 9 | gap junction protein gamma 1, cell adhesion molecule 2 | cadherin 2 |
| rno-miR-322-3p | - | protocadherin beta 10, claudin 4 | - |
| rno-let-7i-5p | integrin subunit alpha L | claudin 12, protocadherin 19 | - |
| rno-miR-199a-3p | fibronectin 1, integrin alpha FG-GAP repeat containing 1 | integrin beta 8, protocadherin 17 | - |
| rno-miR-181a-1-3p | - | integrin beta-like 1 | - |
| rno-miR-493-3p | - | - | - |
| rno-miR-410-3p | cadherin 11, claudin 10 | protocadherin 8, cell adhesion molecule 4 | - |
| rno-miR-1b | gap junction protein, alpha 1, integrin subunit alpha 11 | protocadherin 17, cadherin | - |
| rno-miR-212-3p | integrin subunit beta 1 binding protein 1, claudin 1 | protocadherin 10, cadherin 8 type 2 | - |
| rno-miR-466b-5p | integrin subunit alpha 11 | claudin 7, integrin, alpha E, integrin beta-like 1 | - |
| rno-miR-142-5p | collagen type IV alpha 3 binding protein, occludin, claudin 8 | integrin, alpha 9 | - |
| rno-miR-25-3p | claudin 11, integrin subunit alpha 5 | claudin 11 | claudin 8 |
| rno-miR-181a-5p | - | cadherin 8 type 2, integrin alpha 6, gap junction protein alpha 9 | - |
| rno-miR-126a-5p | afadin, adherens junction formation factor, claudin 8 | occludin, integrin alpha 8, protocadherin 10 | - |
| rno-miR-425-3p | - | integrin beta 3 | - |
| rno-miR-221-3p | claudin domain containing 1, gap junction protein, alpha 1 | protocadherin alpha 3, integrin beta 8 | - |
| rno-miR-652-3p | - | - | - |
| rno-miR-27a-3p | integrin subunit alpha 8, claudin domain containing 1 | protocadherin 9, cadherin 11 type 2, OB-cadherin, integrin alpha 8 | integrin subunit alpha 8 |
| rno-miR-486 | claudin 1 | - | - |
| rno-miR-150-5p | claudin 8 | protocadherin 1 | - |
| rno-miR-133a-3p | integrin subunit alpha 8, tight junction protein 2 | protocadherin 17 | - |
| rno-miR-146a-5p | integrin subunit alpha 6, claudin 19 | - | - |
| rno-let-7d-5p | collagen type 4 alpha 1 chain, collagen type 5 alpha 2 chain, claudin 12 ,integrin subunit alpha L | - | - |
| rno-miR-489-5p | - | claudin 4, cadherin 10 type 2 | - |
| rno-miR-143-3p | integrin subunit alpha 6, collagen type 1 alpha 1 chain, collagen type 5 alpha 3 chain | cadherin 5 type 2 | - |
| rno-miR-301a-3p | - | cadherin 20 type 2, integrin alpha 9 | - |
| rno-miR-378a-3p | - | - | - |
| rno-miR-92a-3p | integrin subunit alpha 5, integrin subunit alpha 8, integrin-binding sialoprotein, claudin 11, claudin 2, collagen type I alpha 2 chain | integrin alpha 5, cell adhesion molecule 2 | - |
| rno-miR-193b-3p | junctional adhesion molecule 3, claudin 23 | - | - |
| rno-miR-139-5p | integrin subunit alpha E, adherens junctions associated protein 1, claudin 6 | cadherin 20, type 2, adherens junctions associated protein 1 | - |
| rno-miR-24-2-5p | gap junction protein, beta 6, claudin 12 | claudin 12, protocadherin beta 16, adherens junctions associated protein 1 | - |
| rno-miR-27b-3p | integrin subunit alpha 8, claudin domain containing | protocadherin 9, cadherin 5 type 2, integrin alpha 5 | - |
| rno-let-7a-5p | integrin subunit alpha L, claudin 12, collagen type 5 alpha 2 chain, collagen type 4 alpha 1 chain | claudin 12, gap junction protein alpha 9, cell adhesion molecule 2 | - |
| rno-miR-183-5p | integrin subunit beta 1, gap junction protein alpha 5, adherens junctions associated protein 1 | integrin, beta 1 , occludin | integrin subunit beta 1 |
| rno-miR-181d-5p | - | cadherin 8, type 2, protocadherin alpha 5, integrin alpha 6, integrin, alpha 1 | - |
| rno-miR-133b-3p | integrin subunit alpha 8 | protocadherin 17, tight junction protein 2 | - |
| rno-miR-218a-2-3p | integrin subunit beta 3 binding protein | claudin domain containing 1, integrin, alpha 9 | - |
| rno-miR-1193-3p | - | - | - |
| rno-let-7e-5p | integrin subunit alpha L, claudin 12, collagen type 5 alpha 2 chain, collagen type 4 alpha 1 chain | claudin 12, protocadherin 19, cadherin 22 type 2 | - |
| rno-miR-205 | afadin, adherens junction formation factor, claudin 8, claudin 11 | claudin 11 | claudin 11 |
| rno-miR-802-5p | claudin 12, claudin 6 | cadherin 11 type 2 | - |
| rno-miR-10b-3p | gap junction protein, alpha 5, gap junction protein, alpha 1, collagen type 1 alpha 1 chain | occludin, integrin beta-like 1 | - |
| rno-miR-148b-5p | gap junction protein alpha 1, claudin domain containing 1 | - | - |
| rno-miR-155-3p | - | catenin | - |
| rno-miR-19a-3p | afadin, adherens junction formation factor, gap junction protein, alpha 1, claudin 8 | integrin, beta 8, gap junction protein alpha 1, | - |
| rno-miR-29a-5p | collagen type 4 alpha 3 binding protein, collagen type 4 alpha 4 chain | junctional adhesion molecule 3, cadherin 7 type 2, protocadherin beta 4 | - |
| rno-miR-3544 | - | occludin, integrin, alpha V, integrin, alpha 1, integrin alpha 7, integrin, beta 8 | - |

**Table S4**

**Tab. 4 . Results of miRs target research in OA rat group.**

| **miRNA** | **Independent databases** | | |
| --- | --- | --- | --- |
|  | **miRDB** | **TargetScan** | **TaRBase** |
| rno-let-7c-5p | claudin 12, cerebral endothelial cell adhesion molecule | **i**ntegrin beta 3, integrin beta 8, claudin 23, protocadherin 19, cadherin 22 type 2, gap junction protein, alpha 9 | **-** |
| rno-miR-17-1-3p | claudin 9, tight junction protein 1 | claudin 22, claudin 23, claudin 1, protocadherin 1, tight junction protein 2, gap junction protein delta 2, adherens junctions associated protein 1, junctional adhesion molecule 3 | - |
| rno-miR-190a-5p | tight junction protein 1, claudin 23 | cadherin 2, type 1, N-cadherin | - |
| rno-miR-212-5p | cadherin 11, cadherin 11 |  | - |
| rno-miR-296-3p | protocadherin beta 6 |  | - |
| rno-miR-148a-5p | gap junction protein, alpha 1 | occludin, integrin, alpha V, integrin, alpha 4, integrin, beta 1 | - |
| rno-miR-148b-3p | integrin subunit alpha 5, gap junction protein, delta 2 | integrin beta 3, claudin 19, occludin, integrin alpha 6 | - |
| rno-miR-466b-3p | cadherin 11, laminin subunit gamma 1 | integrin, alpha V, integrin, alpha 1, claudin 23, claudin 1, protocadherin 10, cadherin 18, type 2, protocadherin beta 16, gap junction protein, beta 3, 31kDa, gap junction protein, alpha 5, 40kDa | - |
| rno-miR-370-3p | tight junction protein 1, protocadherin 19 | gap junction protein, alpha 4, integrin beta 1 | - |
| rno-miR-488-3p | integrin subunit alpha 6, protocadherin 19 |  | - |
| rno-miR-181d-3p | claudin 4 | integrin, alpha 9, claudin 10, claudin 19, protocadherin 17 | - |
| rno-miR-1247-3p |  | integrin, beta 5, integrin, alpha 11, claudin 19, cadherin 19, type 2, protocadherin gamma subfamily B, 3, protocadherin gamma subfamily B, 4, gap junction protein, epsilon 1, 23kDa, gap junction protein, alpha 1, 43kDa, gap junction protein, alpha 9, 59kDa, gap junction protein, beta 1, 32kDa, gap junction protein, gamma 1, 45kDa | - |
| rno-miR-3547 | cerebral endothelial cell adhesion molecule | gap junction protein, beta 4, 30.3kDa, gap junction protein, alpha 4, 37kDa, gap junction protein, delta 2, 36kDa, adherens junctions associated protein 1, protocadherin alpha 1, protocadherin alpha 8, integrin, beta 3 (platelet glycoprotein IIIa, antigen CD61), integrin, alpha 6 | - |
| rno-miR-146a-3p | cadherin 11 | integrin, beta 8, integrin, alpha 8, integrin, beta 3 (platelet glycoprotein IIIa, antigen CD61), occludin, cadherin 20, type 2, adherens junctions associated protein 1, gap junction protein, gamma 2, 47kDa, gap junction protein, alpha 4, 37kDa, gap junction protein, alpha 9, 59kDa, gap junction protein, gamma 1, 45kDa | - |
| rno-miR-3594-5p | - | integrin, beta 4, integrin, beta-like 1 (with EGF-like repeat domains), integrin, alpha L (antigen CD11A (p180), lymphocyte function-associated antigen 1; alpha polypeptide), protocadherin 1, cell adhesion molecule 3, cadherin 24, type 2, gap junction protein, alpha 3, 46kDa | - |
| rno-miR-135a-5p | - | protocadherin 9 | - |
| rno-miR-3075 | - | integrin, alpha 9, integrin, alpha 5 (fibronectin receptor, alpha polypeptide), integrin, alpha 6, gap junction protein, delta 3, 31.9kDa, gap junction protein, gamma 1, 45kDa, adherens junctions associated protein 1 | - |
| rno-miR-429 | cadherin 11, protocadherin 19 | occludin, gap junction protein, gamma 1, 45kDa | - |
| rno-miR-935 | protocadherin alpha 4 | gap junction protein, alpha 1, 43kDa, gap junction protein, alpha 5, 40kDa, integrin, alpha 11, integrin, alpha V | - |
| rno-miR-369-5p | - | integrin, alpha 1, cadherin-related family member 2, claudin 1 | - |
| rno-miR-296-5p | - | - | - |
| rno-miR-410-5p | - | gap junction protein, beta 4, 30.3kDa, adherens junctions associated protein 1, protocadherin 11 X-linked, cadherin 12, type 2 (N-cadherin 2), gap junction protein, beta 4, 30.3kDa | - |
| rno-miR-149-5p | gap junction protein, gamma 2 | protocadherin 19, protocadherin 1 | - |
| rno-miR-1298 | - | - | - |
| rno-miR-16-5p | protocadherin 9, claudin 12 | protocadherin alpha 10, protocadherin alpha 7, protocadherin alpha 5 | - |
| rno-miR-127-3p | - | - | - |
| rno-miR-126a-3p | - | integrin, alpha 6, protocadherin 7 | - |
| rno-let-7f-5p | - | gap junction protein, alpha 9, 59kDa, claudin 12, claudin 23, integrin, beta 8, protocadherin 19 | - |
| rno-miR-25-3p | - | protocadherin 11 Y-linked, protocadherin 20, protocadherin 10, protocadherin 1, integrin, alpha V, integrin, alpha 5 (fibronectin receptor, alpha polypeptide), integrin, alpha 8 | - |
| rno-miR-34b-3p | integrin subunit beta 5 | integrin, beta 8, integrin, alpha V, claudin 1, protocadherin 20 | - |
| rno-miR-376b-3p | integrin subunit beta 5, protocadherin beta 21, claudin 1, cadherin 9 | catenin (cadherin-associated protein), alpha 3, protocadherin 17, integrin, beta 8 | - |
| rno-miR-181a-5p | protocadherin alpha 4 | gap junction protein, alpha 9, 59kDa, cadherin 8, type 2, protocadherin alpha 5, protocadherin alpha 3 | - |
| rno-miR-802-3p | - | claudin 12 | - |
| rno-miR-146a-5p | - | gap junction protein, gamma 1, 45kDa | - |
| rno-miR-222-5p | - | claudin 12, claudin 22, claudin 2, claudin 4 | - |

**Figure S1**

**Fig. S1 MiR-183-5p** **tissue specificity according to Human TissueAtlas** (<https://ccb-web.cs.uni-saarland.de/tissueatlas2/patterns/hsa/mirna/hsa-miR-183-5p/>)

**Figure S2**

**Fig. S2 MiR-122-5p** **tissue specificity according to Human TissueAtlas** (<https://ccb-web.cs.uni-saarland.de/tissueatlas2/patterns/hsa/mirna/hsa-miR-122-5p/>)

**Figure S3**

**Fig. S3 Immunocytochemical staining of primary brain microvascular endothelial cells with endothelial cell marker.**

**Figure S4**

**Fig. S4 Immunocytochemical staining of RBE4 cells with endothelial cell markers.**
